# The Evolution of Vp1 Gene in Enterovirus C Species Sub-Group That Contains Types CVA-21, CVA-24, EV-C95, EV-C96 and EV-C99

**DOI:** 10.1371/journal.pone.0093737

**Published:** 2014-04-02

**Authors:** Teemu Smura, Soile Blomqvist, Tytti Vuorinen, Olga Ivanova, Elena Samoilovich, Haider Al-Hello, Carita Savolainen-Kopra, Tapani Hovi, Merja Roivainen

**Affiliations:** 1 Department of Infectious Disease Surveillance and Control, National Institute for Health and Welfare (THL), Helsinki, Finland; 2 Department of Virology, Haartman Institute, Faculty of Medicine, University of Helsinki, Helsinki, Finland; 3 Department of Virology, University of Turku, Turku, Finland; 4 M.P. Chumakov Institute of Poliomyelitis and Viral Encephalitides, Russian Academy of Medical Sciences, Moscow, Russia; 5 Republican Research and Practical Center for Epidemiology and Microbiology, Minsk, Republic of Belarus; The University of Hong Kong, Hong Kong

## Abstract

Genus *Enterovirus* (Family *Picornaviridae*,) consists of twelve species divided into genetically diverse types by their capsid protein VP1 coding sequences. Each enterovirus type can further be divided into intra-typic sub-clusters (genotypes). The aim of this study was to elucidate what leads to the emergence of novel enterovirus clades (types and genotypes). An evolutionary analysis was conducted for a sub-group of *Enterovirus C* species that contains types Coxsackievirus A21 (CVA-21), CVA-24, Enterovirus C95 (EV-C95), EV-C96 and EV-C99. VP1 gene datasets were collected and analysed to infer the phylogeny, rate of evolution, nucleotide and amino acid substitution patterns and signs of selection. In VP1 coding gene, high intra-typic sequence diversities and robust grouping into distinct genotypes within each type were detected. Within each type the majority of nucleotide substitutions were synonymous and the non-synonymous substitutions tended to cluster in distinct highly polymorphic sites. Signs of positive selection were detected in some of these highly polymorphic sites, while strong negative selection was indicated in most of the codons. Despite robust clustering to intra-typic genotypes, only few genotype-specific ‘signature’ amino acids were detected. In contrast, when *different* enterovirus types were compared, there was a clear tendency towards fixation of type-specific ‘signature’ amino acids. The results suggest that permanent fixation of type-specific amino acids is a hallmark associated with evolution of different enterovirus types, whereas neutral evolution and/or (frequency-dependent) positive selection in few highly polymorphic amino acid sites are the dominant forms of evolution when strains *within* an enterovirus type are compared.

## Introduction

Enteroviruses (genus *Enterovirus*, family *Picornaviridae*) are small non-enveloped positive strand RNA viruses with icosahedral capsid symmetry. Enteroviruses are classified to twelve species, *Enterovirus A* to *H*, *J* and *Rhinovirus A* to *C* [1]. Seven of the species, *Enterovirus A* to *D* (formerly named *Human enterovirus A* to *D*) and *Rhinovirus A* to *C* (formerly named *Human rhinovirus A* to *C*) are known to infect humans. Enteroviruses are associated with a variety of clinical diseases, such as aseptic meningitis, encephalitis, paralytic disease, respiratory infections, and acute haemorrhagic conjunctivitis (AHC) although most enterovirus infections are sub-clinical [2]. Enteroviruses are transmitted mostly via fecal-oral and respiratory routes.

Enterovirus genome contains approximately 7500 nucleotides. The genome consists of a single open reading frame (ORF) that is flanked by 5′ end and 3′ end untranslated regions (5′UTR and 3′UTR). The ORF is translated to a single polypeptide that is autocatalytically cleaved to P1, P2 and P3 polyproteins. The P1 polyprotein is further cleaved to capsid proteins VP4 to VP1, whereas P2 and P3 are cleaved to non-structural proteins 2A–2C and 3A–3D, respectively.

On the basis of the capsid coding P1 region, enteroviruses form genetically highly diverse types that are equivalent to serotypes defined by antigenic properties [3], [4]. Currently, enterovirus types are defined by the sequence divergences in the capsid protein VP1 coding region. Members of the same type have more than 75% nucleotide (nt) and more than 88% amino acid (aa) similarities in the VP1 region [3], [5]. Correspondingly, the strains that have less than 70% nucleotide and 85% amino acid similarities are classified to different types. Empirical observations suggest that, in general, the pair wise sequence similarities between enterovirus strains do not form a continuum across the type designations, but rather the members of different types are clearly separated [3]. However, divergent strains that have pairwise nt/aa similarities in the ‘grey-zone’ of current typing (i.e. 70–75% nt and/or 85–88% aa similarity), such as some CVA-24 and EV-C99 strains, are detected regularly [5].

Although the current typing standard is of great practical value, little is known about the evolutionary processes behind the observed sequence similarity patterns. Likewise, although the implementation of molecular methods for genetic enterovirus typing has led to the discovery of a large number of novel enterovirus types (reviewed in [6]), much less is known about evolutionary processes behind the formation of the enterovirus types.

The aim of this study was to elucidate the evolutionary processes behind the origin and emergence of novel enterovirus clades (types and genotypes).The clearly resolved hierarchical phylogeny of EV-C species [5], [7], [8] provides an opportunity to study the evolutionary patterns that have led to the emergence of novel enterovirus clades. In this study, an evolutionary analysis was conducted for a sub-group of EV-C species that contains types CVA-21, CVA-24, EV-C95, EV-C96 and EV-C99. Of these enterovirus types, CVA-21 induces mainly mild respiratory infections [9], [10], whereas EV-C96, EV-C99 and some of the (prototype-like) CVA-24 strains have been isolated from faecal samples of healthy individuals and patients with paralytic disease [5], [8], [11]–[20]. A distinct lineage of CVA-24, so-called variant strains (CVA-24v), induce acute haemorrhagic conjunctivitis (AHC) [21] and are transmitted via direct or indirect contact with eye-secretions [2]. EV-C95 is a recently discovered enterovirus type (Helene Norder unpublished [1]. So far, only two strains of this type have been characterized [17]. Parallel datasets of VP1 sequences were collected and analysed to infer the phylogenetic structure, rate of evolution, nucleotide and amino acid substitution patterns and signs for selection in the VP1 coding region.

## Material and Methods

### Ethics statement

The virus samples were collected with the consent of The Institutional Review Board of National Institute for Health and Welfare (THL), the Ethics Committee of M.P. Chumakov Institute of Poliomyelitis and Viral Encephalitides of Russian Academy of Medical Sciences and the Ethical Committee of the Minsk Municipality and analyzed anonymously. The sewage samples were obtained from Viikki wastewater treatment plant in Helsinki, Finland. No specific permission was required for the surveillance for enteroviruses from sewage. The field studies did not involve endangered or protected species.

### Viruses

The virus strains isolated in this study are listed in Table 1. Virus strains were isolated from sewage samples collected during environmental surveillance for polioviruses in Finland using a two-phase concentration method [22]. In addition, clinical enterovirus isolates sent to the national enterovirus reference laboratory (National Institute for Health and Welfare, THL) from other Finnish laboratories were included in the study [23]. The rest of the virus strains were received as untypeable nonpolio enteroviruses (NPEV) from a number of National Polio Laboratories of the WHO Polio Laboratory Network supporting the Global Poliovirus Eradication Initiative. Human rhabdomyosarcoma (RD), human colorectal adenocarcinoma (CaCo-2), human cervical carcinoma (HeLa) and green monkey kidney (GMK) cell lines were used for virus isolation.

**Table 1:** The strains sequenced in this study.

Strain	Collection Date	Country	Sample Type
CVA-21-EST06-E1783-20171_28-Feb-2006	28-Feb-2006	Estonia	Sewage
CVA-21-FIN03-862-36252	2003	Finland	Stool
CVA-21-FIN06-E1906-28163_27-Sep-2006	27-Sep-2006	Finland	Sewage
CVA-21-FIN06-E2006-32088_19-Dec-2006	19-Dec-2006	Finland	Sewage
CVA-21-FIN06-EV06-34A-30796_14-Nov-2006	14-Nov-2006	Finland	Stool
CVA-21-LVA03-756_5-Mar-2003	5-Mar-2003	Latvia	
CVA-21-RUS01-15341_4-Jul-2001	4-Jul-2001	Russia	Stool
CVA-21-SVK05-E1571_Skalica_17-Feb-2005	17-Feb-2005	Slovak Republic	Sewage
CVA-21-SVK07-E2058_ Dunajska Streda_10-Jan-2007	10-Jan-2007	Slovak Republic	Sewage
CVA-24-AUT05-1600_12-Apr-2005	12-Apr-2005	Austria	
CVA-24-FIN02-671	2002	Finland	Sewage
CVA-24-FIN04-EV04-27A-2124	2004	Finland (India)	Stool
CVA-24-FIN04-EV04-34A-3787	2004	Finland (India)	Stool
CVA-24-FIN05-1-7920-2005	2005	Finland (China)	Stool
CVA-24-FIN05-1663-13794_18-Oct-2005	18-Oct-2005	Finland	Stool
CVA-24-FIN05-EV05-17A_31-Oct-2005	31-Oct-2005	Finland (China)	Stool
CVA-24-FIN06-1869-25202_6-Sep-2006	6-Sep-2006	Finland	Stool
CVA-24-FIN06-EV06-32A-30807_24-Oct-2006	24-Oct-2006	Finland	Stool
CVA-24-FIN06-EV07-2A-29392_18-Dec-2006	18-Dec-2006	Finland	Stool
CVA-24-FIN07-EV07-12A-33320_15-May-2007	15-May-2007	Finland	Stool
CVA-24-FIN07-EV07-13A-33425_28-May-2007	28-May-2007	Finland	Stool
CVA-24-FIN07-EV07-25A-37012_22-Aug-2007	22-Aug-2007	Finland	Stool
CVA-24-FIN07-EV07-26A-37474_30-Aug-2007	30-Aug-2007	Finland	Stool
CVA-24-FIN07-EV07-42A-51242_2007	2007	Finland	Stool
CVA-24-FIN07-EV08-1A-51415_18-Dec-2007	18-Dec-2007	Finland	Stool
CVA-24-FIN08-EV08-28A-59927_10-Oct-2008	10-Oct-2008	Finland	Stool
CVA-24-FIN08-EV08-3A-51551_30-Jan-2008	30-Jan-2008	Finland	Stool
CVA-24-FIN09-2996-72114_20-May-2009	20-May-2009	Finland (India)	Stool
CVA-24-FIN09-3137-78084_23-Oct-2009	23-Oct-2009	Finland	Stool
CVA-24-FIN09-3140-78104_22-Oct-2009	22-Oct-2009	Finland	Stool
CVA-24-FIN09-EV09-11B-70968_14-May-2009	14-May-2009	Finland	Stool
CVA-24-FIN09-EV09-12B-70971_5-May-2009	5-May-2009	Finland	Stool
CVA-24-FIN09-EV09-14A-72174_15-Jun-2009	15-Jun-2009	Finland	Stool
CVA-24-FIN09-EV09-7A-70636_27-Feb-2009	27-Feb-2009	Finland	Stool
CVA-24-FIN10-E3319-A341_23-Mar-2010	23-Mar-2010	Finland	Sewage
CVA-24-FIN10-EV10-23B-91082_30-Aug-2010	30-Aug-2010	Finland	Stool
CVA-24-FIN10-EV10-2B_2-Mar-2010	2-Mar-2010	Finland	Stool
CVA-24-LVA03-757_19-Jun-2003	19-Jun-2003	Latvia	
CVA-24-RUS-00-14038_21-Nov-2000	21-Nov-2000	Russia	Stool
CVA-24-RUS-01-14455_14-Feb-2001	14-Feb-2001	Russia	Stool
CVA-24-RUS-TKM01-14868_10-Mar-2001	10-Mar-2001	Russia (Turkmenistan)	Stool
CVA-24-RUS-KGZ01-15071_18-Jun-2001	18-Jun-2001	Russia (Kyrgyzstan)	Stool
CVA-24-RUS-UZB01-15213_20-Jun-2001	20-Jun-2001	Russia (Uzbekistan)	Stool
CVA-24-RUS-TKM01-15327_23-Jun-2001	23-Jun-2001	Russia (Turkmenistan)	Stool
CVA-24-RUS-KGZ01-15876_20-Jul-2001	20-Jul-2001	Russia (Kyrgyzstan)	Stool
CVA-24v-FIN07-EV07-20A-34537_15-Jul-2007	15-Jul-2007	Finland	Conjunctival secretion
CVA-24v-FIN07-EV07-22B_13-Aug-2007	13-Aug-2007	Finland	Stool
EV-C96-FIN08-EV08-10A-55557_14-May-2008	14-May-2008	Finland	Stool
EV-C96-FIN09-2983-70820_28-May-2009	28-May-2009	Finland	Stool
EV-C96-FIN09-EV09-13B_5-May-2009	5-May-2009	Finland	Stool
EV-C96-FIN09-EV09-9A-70153_24-Mar-2009	24-Mar-2009	Finland	Stool
EV-C96-FIN10-EV10-3A-84622_27-Apr-2010	27-Apr-2010	Finland	Stool
EV-C96-FIN12-EV12-11B_9-May-2012	9-May-2012	Finland	Stool
EV-C99-FIN06-EV06-31B-30779_6-Nov-2006	6-Nov-2006	Finland	Stool
EV-C99-FIN07-EV07-24A-37056_17-Aug-2007	17-Aug-2007	Finland	Stool
EV-C99-FIN07-EV08-2A-51416_20-Dec-2007	20-Dec-2007	Finland	Stool
EV-C99-FIN08-EV08-19A-56305_11-Aug-2008	11-Aug-2008	Finland	Stool
EV-C99-FIN08-EV08-7A-55125_7-Apr-2008	7-Apr-2008	Finland	Stool
EV-C99-RUS-TKM00-13831_8-Sep-2000	8-Sep-2000	Russia (Turkmenistan)	Stool
EV-C99-SVK03-23-20226_7-Feb-2003	7-Feb-2003	Slovak Republic	
EV-C99-SVK04-E1152-44722_19-May-2004	19-May-2004	Slovak Republic	Sewage
EV-C99-FIN09-EV09-15A-72176_22-Jun-2009	22-Jun-2009	Finland	Stool
EV-C99-FIN11-4266-10449_6-Oct-2011	6-Oct-2011	Finland	Stool
EV-C99-FIN09-2991-70979_2-Jun-2009	2-Jun-2009	Finland (Nigeria)	Stool
EV-C99-BLR00-32864-2000	2000	Republic of Belarus	Stool
EV-C99-BLR00-32878-2000	2000	Republic of Belarus	Stool
EV-C99-BLR00-32881-2000	2000	Republic of Belarus	Stool
EV-C99-BLR00-32887-2000	2000	Republic of Belarus	Stool
EV-C99-BLR00-33291-2000	2000	Republic of Belarus	Stool
EV-C99-BLR00-33305-2000	2000	Republic of Belarus	Stool
EV-C99-BLR00-33405-2000	2000	Republic of Belarus	Stool
EV-C99-BLR00-33483-2000	2000	Republic of Belarus	Stool

### Partial VP1 RT-PCR and sequencing

Viral RNA was extracted from infected cell cultures with RNeasy Total RNA kit (Qiagen, Hilden, Germany) or E.Z.N.A. Total RNA Kit Omega (Bio-Tek Inc., Doraville, GA, USA) according to the manufacturer's instructions. RT-PCR was carried out as described previously [24] using primers 292 and 222. PCR amplicons were purified with the QIAquick gel extraction kit (Qiagen). Sequencing reactions with BigDye Terminator cycle sequencing ready reaction kit v3.1 (Life Technologies, Carlsbad, CA, USA) and sequencing with ABI3730 Automatic DNA Sequencer (Life Technologies) were performed by the Institute for Molecular Medicine Finland (FIMM) Sequencing Laboratory. The electropherograms were analysed using Geneious Pro 6.0 software (Biomatters Ltd, Auckland, New Zealand, http://www.geneious.com).

### Sequence dataset collection

Two parallel datasets were constructed from the sequences. The first dataset contained partial VP1 sequences characterized in this study (N = 72) (Table 1), the overlapping CVA-21, CVA-24, EV-C95, EV-C96 and EV-C99 sequences retrieved from the GenBank and the overlapping sequences of the prototype strains of other EV-C types. Altogether this dataset consisted of 264 sequences. The consensus alignment of partial VP1 dataset consisted of 343 nucleotide sites. The second dataset contained the strain CVA-24-FIN05-1-7920 and all complete CVA-21, CVA-24, EV-C95, EV-C96 and EV-C99 VP1 sequences found in the GenBank (27.2.2013). The complete VP1 dataset consisted of 97 strains. The consensus alignment of this dataset contained 927 nucleotides. To relieve computational demands and correct data collection bias, only one representative sequence from the clusters that shared more than 99% similarity with each other was included in the analysis.

### Sequence analysis

The sequences were aligned using ClustalW algorithm (for codons) implemented in MEGA version 5.05 [25] followed by manual refinement. Phylogenetic trees were constructed using neighbor-joining (NJ) method implemented in MEGA version 5.05 and Bayesian Monte Carlo Markov Chain (MCMC) method implemented in BEAST version 1.7.4 [26]. For NJ-trees, bootstrap resampling with 1000 replicates was conducted. Various substitution models including Tamura-Nei (TN93) [27] and general time reversible (GTR) [28] were utilized.

The rates of evolution and divergence times of the virus lineages were estimated using Bayesian MCMC method implemented in BEAST version 1.7.4 [26]. Different molecular clock and demographic models were compared by calculating the marginal likelihoods of the data [29]. Bayes factors (BF) were calculated for each pair of models with Tracer 1.5 [30]. The uncorrelated relaxed molecular clock approach (log-normal distribution fitted the data significantly better than strict clock or the relaxed molecular clock approach with exponential distribution (log BF>400). The Bayesian skyline demographic model fitted the data significantly better than constant or exponential models (log BF>400). Therefore, the analyses were performed using a relaxed molecular clock model (the uncorrelated log-normal distributed model) [31], GTR model of substitution and Bayesian skyline demographic model. The Bayesian analyses were run for 100 million states and sampled every 1000 states. The analyses were carried out on the Bioportal server, University of Oslo (www.bioportal.uio.no) [32] and in CSC – IT Center for Science Ltd. (Espoo, Finland). The analyses were run in duplicate and the log-files were combined to increase the effective sample size. Posterior probabilities were calculated with a burn-in of 10 million states and checked for convergence using Tracer version 1.5. [30].

Codon-specific synonymous (dS) to non-synonymous (dN) rates were estimated using single likelihood ancestor counting (SLAC), fixed effects likelihood (FEL), [33] and FUBAR [34] methods implemented in Datamonkey website (www.datamonkey.org) [35]. For this analysis, the nucleotide substitution model was selected using model test implemented in the Datamonkey website and the phylogenetic trees were reconstructed using the Neighbour-Joining method.

The signatures of directional selection were sought using McDonald–Kreitman test (MK-test) [36] implemented in DnaSP v.5.10 [37] and modified MK-tests [38], [39] implemented in The standard and generalized MKT website (http://mkt.uab.es/mkt/) [38] and Adapt-A-Rate v.1.0 software [40]. The MK-test compares the ratio of fixed non-synonymous to synonymous differences between two predefined groups and the ratio of polymorphic non-synonymous to synonymous differences and assumes that under neutral evolution these ratios should be equal. In the modification by Egea et al., [38] the estimated number of mutations instead of the number of sites in each class are counted. The modification by Bhatt et al., [39] utilizes proportional counting algorithm in which proportional fixation, polymorphic, silent and replacement “site scores” are utilized instead of unambiguously assigned numbers of sites in each class. The modified MK-tests were run independently either including or excluding potentially mildly deleterious low-frequency variants (with <0.05 frequency). The modified test by Bhatt et al., [39] was also run using polymorphic (neutral) classes with frequencies 0-0.50 [41] and 0.05-0.75 (i.e. excluding both potentially mildly deleterious low-frequency variants and highly polymorphic sites that are potentially under frequency-dependent antigenic selection).

The locations of amino acid substitutions were estimated with 3-dimensional structure model of Coxsackievirus A21 pentamer (PDB ID: 1Z7Z) [42] using Jmol [43].

### GenBank accession numbers

The GenBank accession numbers for the sequenced strains are KF128985 - KF129056.

## Results

### Phylogeny and sequence diversities of partial vp1 sequences

To gain insight into the evolution and epidemiology of enterovirus VP1 coding sequences, partial VP1 coding regions of six EV-C96 strains, 19 EV-C99 strains, nine CVA-21 strains, 36 CVA-24 strains and two CVA-24v strains that were isolated during poliovirus surveillance were sequenced and subjected to phylogenetic analysis together with overlapping sequences retrieved from the GenBank (search 27.2.2013).

**Figure 1:**
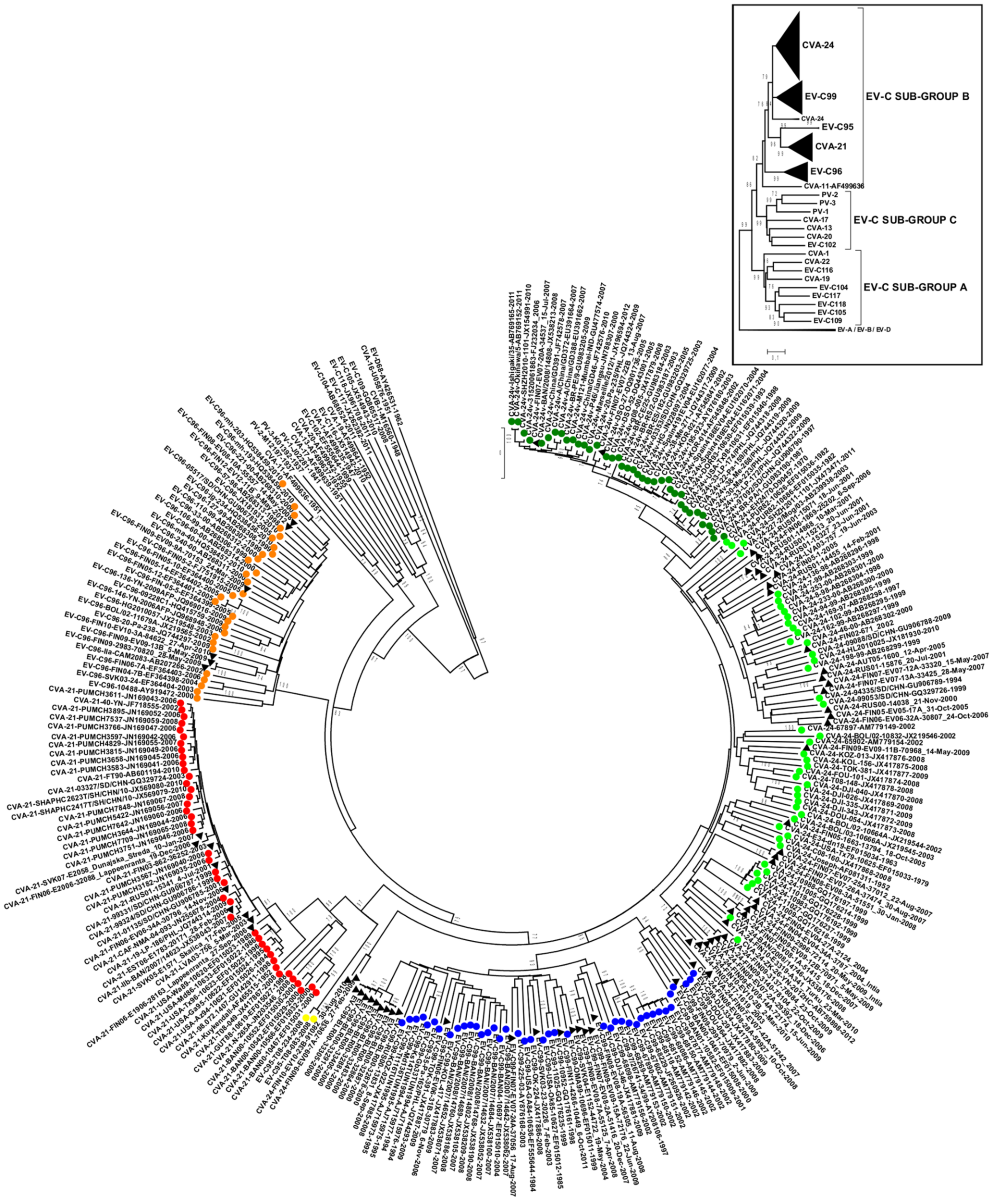
The phylogeny of partial VP1 sequences of EV-C species, including strains isolated in this study. The phylogenetic tree was constructed from partial VP1 coding region (consensus alignment 343 nucleotides) of CVA-21 (red), CVA-24 (green), EV-C95 (yellow), EV-C96 (orange) and EV-C99 (blue) strains and the prototype strains of other EV-C types. The strains sequenced in this study are indicated with black triangles. The tree was constructed using the Neighbour-Joining method and the Tamura-Nei substitution model. The bootstrap support values were calculated for 1000 replicates. The bootstrap support values >70 are shown. The inset represents the same tree with EV-C subgroups A to C identified.

Consistently with the previous studies [5], [7], [44], [45], in the VP1 coding region, all of the EV-C types clustered into three sub-groups, which were designated here as A (CVA-1, CVA-19, CVA-22, EV-C104, EV-C105, EV-109, EV-C116, EV-C117 and EV-C118), B (EV-C95, EV-C96, EV-C99, CVA-21 and CVA-24) and C (CVA-13, CVA-17, CVA-20, EV-C102, PV-1, PV-2 and PV-3) (Fig. 1), with the branching order of sub-group A diverging first from the common ancestor of sub-groups B and C. CVA-11 grouped together with the strains of sub-groups B or C depending on the method of phylogenetic tree construction.

**Figure 2:**
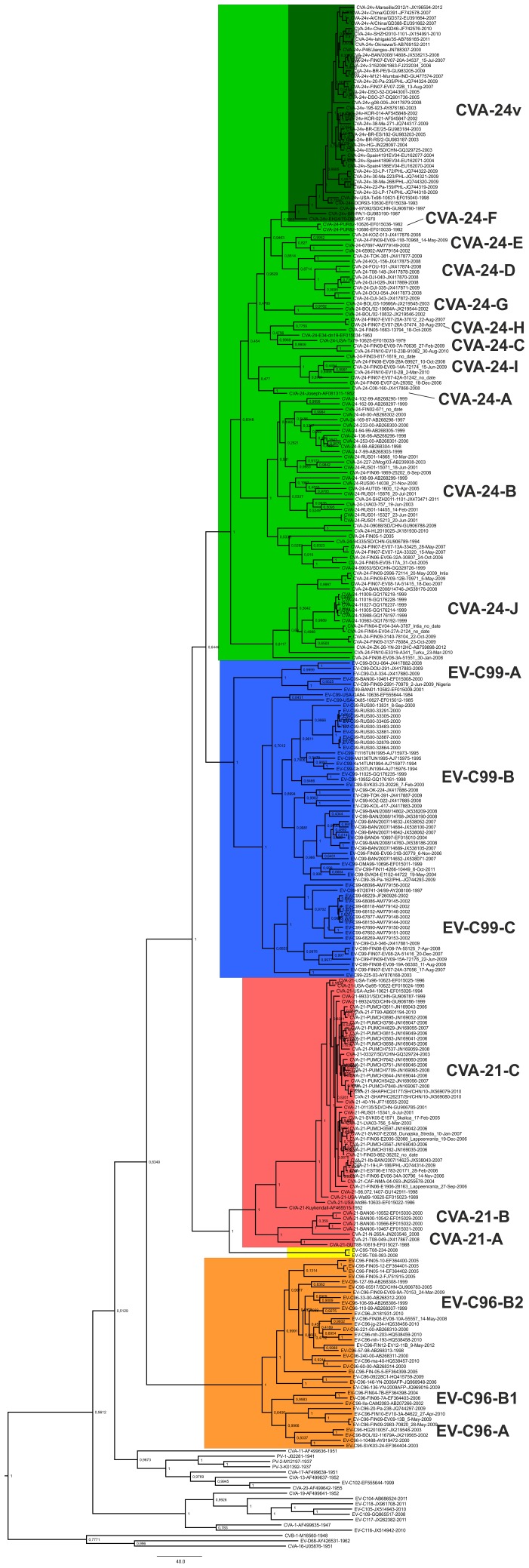
Maximum clade credibility tree of partial VP1 sequences of EV-C species, including strains isolated in this study. The maximum clade credibility tree was constructed from partial VP1 coding region (consensus alignment 343 nucleotides) of CVA-21 (red), CVA-24 (green), EV-C95 (yellow), EV-C96 (orange) and EV-C99 (blue) strains and the prototype strains of other EV-C types. The phylogenetic tree was constructed using Bayesian MCMC method with GTR model of substitution and Bayesian skyline demographic model. Posterior probabilities are shown in each node.

Within EV-C sub-group B, the phylogenetic analysis suggested well-supported hierarchical branching orders. EV-C96 formed an outgroup to the EV-C95/CVA-21/CVA-24/EV-C99 cluster, suggesting that EV-96 has diverged first from a common ancestor of EV-C sub-group B. This divergence has been followed by the branching of the ancestor of EV-C95/CVA-21 from the ancestor of CVA-24/EV-C99 and finally splitting of the ancestors of EV-C95/CVA-21 and CVA-24/EV-C99 into distinct types. Each of the types in EV-C sub-group B were further divided into intra-typic sub-clusters (or genotypes) with robust (i.e. >70) bootstrap support (Fig 1) or with high posterior probability (Fig 2). These genotypes are designated with upper case letters in Figure 2.

Generally similar branching patterns were observed using both NJ (Fig 1.) and the Bayesian MCMC methods (Fig 2). However, there was slight variation in the intra-typic sub-clustering pattern between the methods. In the NJ-tree, the non-AHC-causing CVA-24 strains formed a loose paraphyletic group with low bootstrap support (Fig 1), whereas in MCC tree, all CVA-24 strains formed a monophyletic group with high posterior probability (Fig 2). Many of the intra-typic clusters of EV-C96 and CVA-24 were detected in the MCC-tree (Fig 2) but not in the NJ-tree (Fig. 2). These tentative genotypes were designated as CVA-24-A to J and EV-C96 A to C, respectively (Figure 2). The AHC-causing variant strains of CVA-24 (CVA-24v) formed a monophyletic sub-cluster with both methods. All sub-clusters of CVA-21 were highly supported by both methods. However, a common ancestor for the genotypes CVA-21-A and –B was suggested by the Bayesian method only. For EV-C99 both methods suggested similar branching pattern. In addition, both methods suggested a common ancestor for the previously designated EV-C99 genotypes B and C [17], [46].

**Figure 3:**
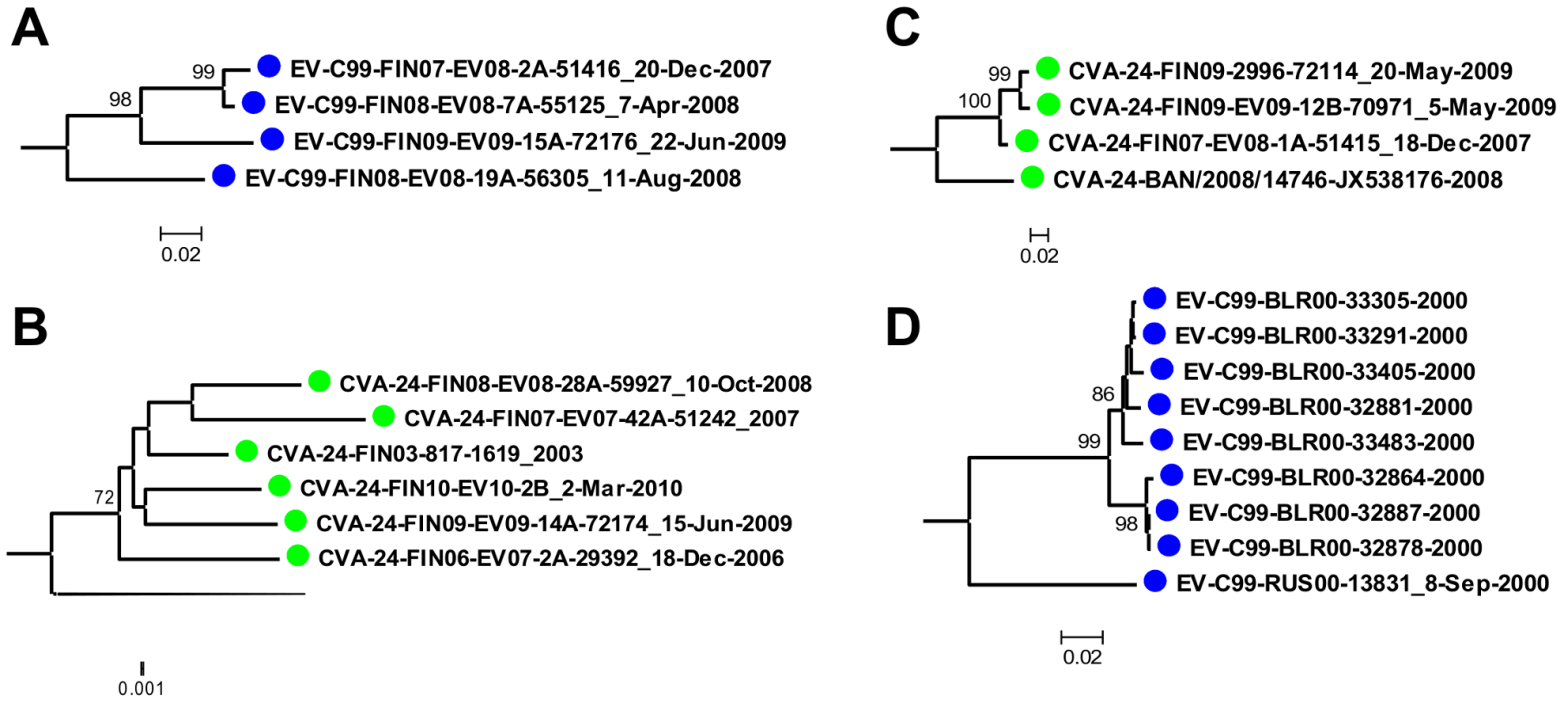
Sub-trees of strains that showed geographic clustering. The phylogenetic trees were constructed from partial VP1 coding region (343 nucleotides) using Neighbour-Joining method and the Tamura-Nei substitution model. Bootstrap values >70 are shown.

Representatives of all EV-C96 and EV-C99 intra-typic genotypes and nearly all CVA-24 genotypes (excluding clusters genotypes A, F and G) were detected in this study. For CVA-21, all the strains sequenced in this study clustered to genotype C. Most of the strains sequenced in this study showed no evidence of geographic or temporal clustering. However, a distinct lineage of EV-C99 was detected in December 2007, April 2008, August 2008 and June 2009 in Finland (Fig 3a). Likewise, a distinct lineage of CVA-24 (designated as CVA-24-I in Fig 2) was detected repeatedly between years 2003 and 2010 (and every year between 2006 and 2010) in Finland (Fig 3b). Another CVA-24 lineage was detected in years 2007 and 2009 in Finland (Fig 3c). This lineage was related to a strain isolated in Bangladesh in 2008 [19]. In addition, eight strains of EV-C99 isolated in an orphanage in Minsk, Belarus during an oral poliovirus vaccination study [47] formed a separate cluster in the phylogenetic analysis (Fig 3d).

A wide variation in the nucleotide and amino acid sequences of the VP1-coding region was observed within each of the EV-C sub-group B types (Table 2) and, between some of the strains, the intra-typic similarities exceeded the 75/85% nucleotide/amino acid identity limit for classification into a single type. The inter-typic nucleotide and amino acid similarities between EV-C99 and CVA-24 were 68.0-81.3% and 78.8–94.9%, respectively. Thus, in agreement with the previous studies [5], although both of these viruses formed clearly separated monophyletic groups, all pair wise comparisons between the strains of these types did not reach the limit of grouping into different types.

**Table 2:** Nucleotide (lower left) and amino acid (upper-right) similarities (%) between VP1 sequences of EV-C sub-group B serotypes.

		EV-C96	EV-C95	CVA-21	EV-C99	CVA-24
**Partial VP1**	**EV-C96**	**71.9/86.2**	62.6–73.5	60.4–70.8	70.0–82.8	70.0–82.9
	**EV-C95**	56.4–64.1	**98.0/99.3**	80.1–86.1	66.4–76.2	67.6–75.7
	**CVA-21**	53.3–66.3	66.5–71.7	**74.1/87.5**	65.7–76.7	66.4–78.6
	**EV-C99**	60.5–72.5	58.7–66.3	58.8–69.3	**71.1/84.0**	78.8–94.9
	**CVA-24**	59.9–73.8	59.5–68.0	61.3–72.5	68.0–81.3	**71.8/84.1**
**Complete VP1**	**EV-C96**	**75.3/89.3**	70.1–71.8	66.4–70.8	72.9–80.9	71.5–77.4
	**EV-C95**	61.5–64.5	**98.0/99.3**	82.9–84.2	73.7–77.5	73.8–77.2
	**CVA-21**	61.0–65.8	68.3–71.5	**76.1/91.9**	72.7–76.8	73.2–78.5
	**EV-C99**	65.1–71.5	64.2–67.5	64.2–69.9	**73.1/86.1**	80.4–88.7
	**CVA-24**	63.6–70.5	64.3–68.7	65.1–70.5	69.8–75.9	**75.0/88.2**

Minimum nucleotide/amino acid similarity within each type is shown in the diagonal (bolded).

### Codon-specific substitution patterns and selection

**Figure 4:**
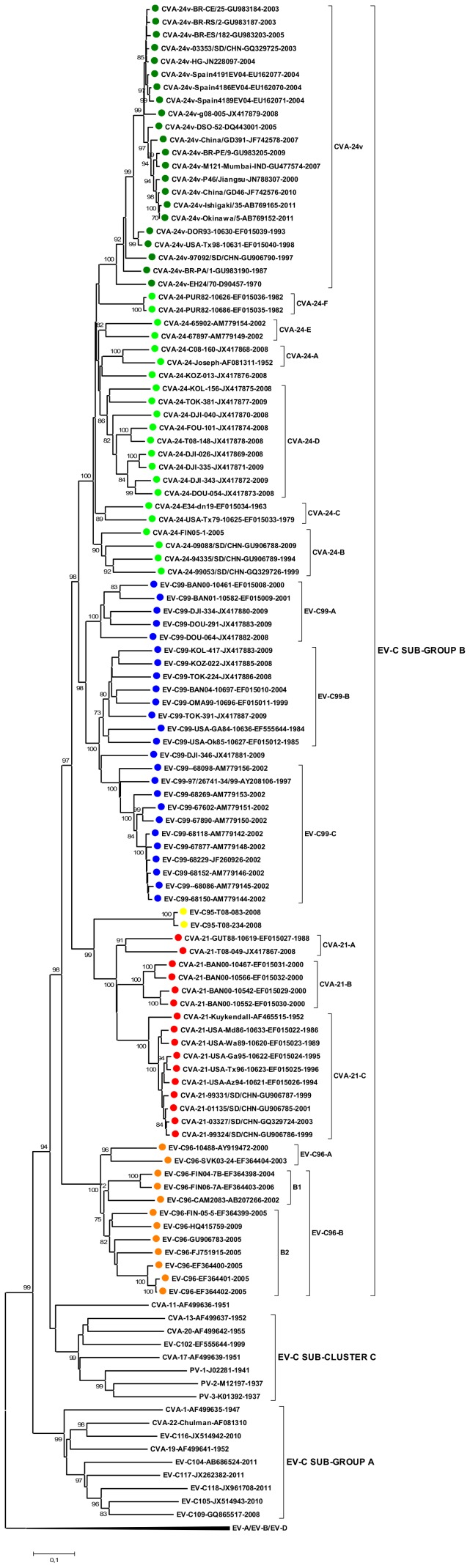
The phylogeny of EV-C species VP1 gene. The phylogenetic tree was constructed from complete VP1 coding region (consensus alignment 927 nucleotides) of those CVA-21 (red), CVA-24 (green), EV-C95 (yellow), EV-C96 (orange) and EV-C99 (blue) strains, of which a complete VP1 sequence was available (GenBank search 27.2.2013), and the prototype strains of other EV-C types. The tree was constructed using the Neighbour-Joining method and the Tamura-Nei substitution model. The bootstrap support values were calculated for 1000 replicates. The bootstrap support values >70 are shown.

In order to elucidate the evolutionary forces leading to the observed phylogeny, a second dataset that contained the strain CVA-24-FIN05-1-7920 and all complete CVA-21, CVA-24, EV-C95, EV-C96 and EV-C99 VP1 sequences found from the GenBank (search 27.2.2013) was analysed. The major EV-C sub-groups A, B and C, as well as the branching order within EV-C sub-group B, were congruent with those observed in the partial VP1 data (Fig 4). Representatives of all intra-typic genotypes of designated using partial VP1 dataset, excluding CVA-24 genotypes G to J, were included in this dataset. The range of intra and inter-typic nucleotide and amino acid similarities were slightly lower in the complete VP1 dataset compared to those observed in the partial VP1 dataset (Table 2).

The VP1 consensus alignment of EV-C96, EV-C95, CVA-21, CVA-24 and EV-C99 contained 309 codons. In comparison to EV-C96, the VP1 amino-terminal domains of CVA-21, CVA-24, EV-C95 and EV-C99 contained deletions of 9, 4, 9 and 5-6 amino acids corresponding to sites 21-29 in the alignment. EV-C99 showed intra-typic differences in the length of the deletion, since the strains of EV-C99 group A had a deletion of 5 amino acids whereas the other strains of EV-C99 had deletions of 6 amino acids. Furthermore, the amino acid sequences of EV-C99 group A and group B/C were highly divergent in this region. The strains of EV-C99 group A also had another deletion of a single amino acid corresponding to site 152 in the consensus alignment. CVA-21 and EV-C95 had a deletion of two amino acids corresponding to sites 103–104 in the consensus alignment.

**Figure 5:**
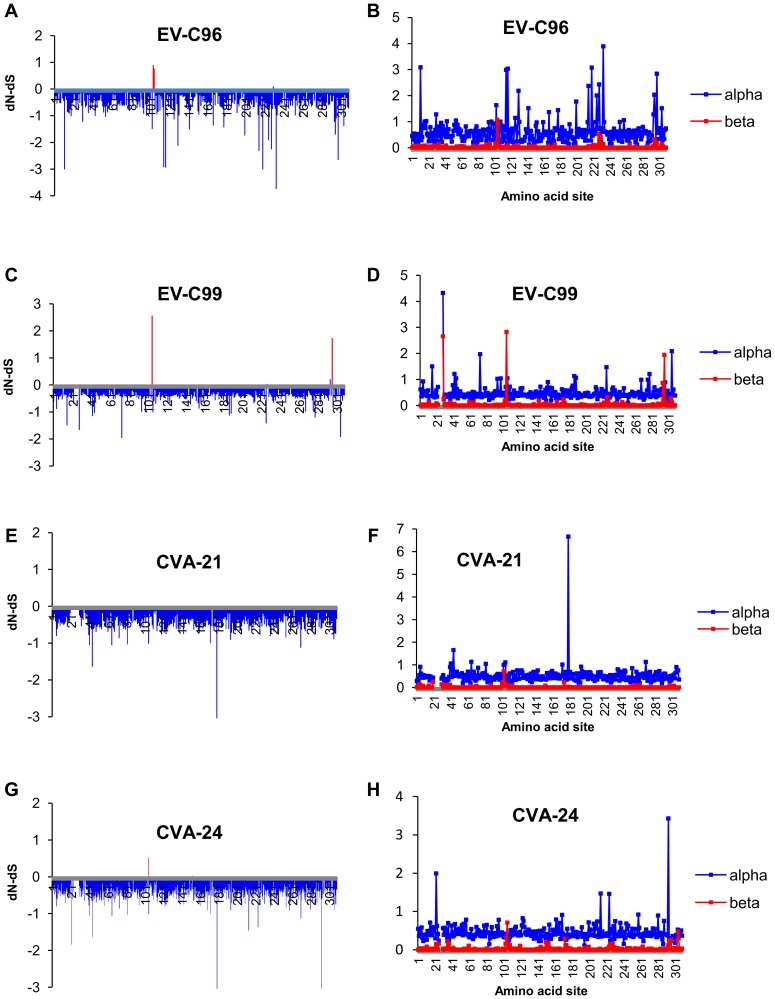
Codon-specific selection. The codon-specific differences between non-synonymous and synonymous rates (normalised dN-dS) (a,c,e,g) and the codon specific posterior distribution means of the synonymous (alpha) and non-synonymous (beta) substitution rates (b,d,f,h) estimated using FUBAR-method. Codons with statistically significant (posterior probability >0.9) evidence for positive selection are shown in red (a, c, e, g).

**Figure 6:**
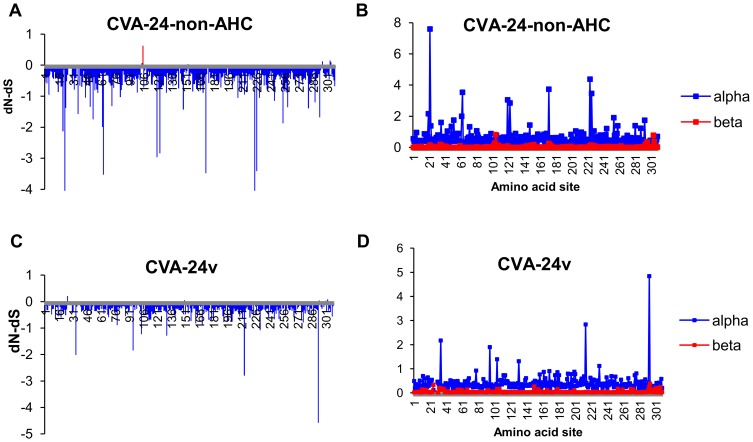
Codon-specific selection in CVA-24v and non-AHC-causing strains of CVA-24. The codon-specific differences between non-synonymous and synonymous rates (normalised dN-dS) (a,c) and the codon specific posterior distribution means of the synonymous (alpha) and non-synonymous (beta) substitution rates (b,d) estimated using FUBAR-method. Codons with statistically significant (posterior probability >0.9) evidence for positive selection are shown in red (a, c).

Codon-specific non-synonymous to synonymous substitution frequencies were assessed using single likelihood ancestor counting (SLAC), fixed effects likelihood (FEL) and FUBAR methods available at the Datamonkey facility (www.datamonkey.org) [35]. The synonymous and non-synonymous substitution frequencies and the normalized dS-dN estimated using FUBAR method are shown in Fig 5. The analyses suggested that strong negative selection was occurring over most of the codons of the VP1-coding region in all of the types. However, elevated dN to dS ratios were detected for some codons by all of the methods, and statistically significant evidence for positive selection was suggested for codons 104 and 105 in EV-C96, codons 105 and 296 in EV-C99 and codon 105 in CVA-24 by at least one of the methods (Table 3). When the AHC-causing variant strains of CVA-24 (i.e. CVA-24v) and other CVA-24 strains were treated as separate groups, evidence for positive selection was detected in different sites (Fig 6). Elevated dN/dS was detected in codons 25 and 150 for CVA-24v and in codon 105 for non-AHC inducing CVA-24 (Table 3). Notably, CVA-24v strains showed strict conservation of alanine at site 105 (only one CVA-24v strain had aspartic acid in this site) whereas the non-AHC-causing strains of CVA-24 had high amino acid polymorphism (i.e. E, D, V, G, R, S, Q, N or T) in this site. Generally, the codons with signs of positive selection were highly polymorphic at amino acid level within the respective type (Table 3). Parallel evolution was also commonly detected in these sites (i.e. similar amino acid substitution had apparently occurred independently in different genetic lineages).

**Table 3:** The VP1 codons that had signs of positive selection.

		Normalized dN-dS			Amino acid composition of the site
Type	Codon	SLAC	FEL	FUBAR	
**EV-C96**	**104**	0.89	2.43	**0.89**	S, T, N, A, V, L, M
	**105**	**1.86**	**3.84**	**0.76**	E, S, T, A, V, I,
**EV-C99**	**105**	0.36	**3.30**	**2.57**	R, K, E, S, T, N, Q, G, P, I, A, V
	**296**	0.42	**4.57**	**1.73**	S, T, A
**CVA-24**	**105**	0.29	**0.57**	**0.52**	R, K, D, E, S, T, N, Q, G, A, V
**CVA-24 (non-AHC)**	**105**	2.63	**1.04**	**0.45**	R, K, D, E, S, T, N, Q, G, A, V
**CVA-24v**	**25**	1.76	**3.36**	0.21	H, S, P, L
	**150**	2.03	**1.76**	0.32	S, T, A

The normalized dN-dS values with statistically significant evidence of positive selection are shown in boldface. The significance level (p-value) of 0.1 or posterior probability of 0.9 were used as confidence limits.

Within each type, most of the non-synonymous substitutions concentrated in distinct regions (Fig 5) that were most likely (on the basis of CVA-21 structure [42]) located at the structurally disordered N- and C-terminal regions and the loops between beta-sheets (Figure S1). Although the codons with high rate of non-synonymous substitutions overlapped partially between the types, at several regions, there were differences between types both in the location of polymorphic amino acid sites and the frequency of amino acid substitutions in these sites. The disparities in the locations of polymorphic sites between EV-C96, EV-C99 and non-AHC causing CVA-24 strains were subtle, polymorphic sites being often dislocated only by few amino acids, whereas more pronounced differences were detected between CVA-21, CVA-24v and other types. For example, the amino acid sites 223-230 were highly polymorphic among EV-C96, EV-C99 and non-AHC causing CVA-24 strains but conserved completely within CVA-21 (LEGENTDA) and CVA-24v (LKDETVS). Notably, several amino acid sites that were polymorphic among non-AHC causing CVA-24 sites were conserved among CVA-24v strains. Such sites included codons 22 (N, Q, K, S vs. Q), 103 (I, T, N, S, E vs. T), 172 (Q, T, I, R, K vs. R), 174 (A, N, T, K vs. T), 295 (N, D, S, A, G, E vs. D), 296 (S, A, E, L, T vs. S) and 304 (A, E, N, K, T, S, D vs. E).

Signs of directional selection between CVA-21, CVA-24, EV-C95, EV-C96 and EV-C99 and between the intra-typic genotypes within these enterovirus types were sought using McDonald-Kreitman test (MK-test) [36] and modifications of this test [38], [39]. The results of the MK-tests are summarized in Table 4. The numbers of sites/substitutions in each MK-test class for different EV-types and intra-typic genotypes are shown in Tables S1–S6. The MK-tests suggested significantly increased proportion of non-synonymous mutations among the fixed sites for all inter-typic comparisons. Likewise, the comparison between EV-C99 groups A and B/C suggested statistically significant evidence of higher than expected number of fixed non-synonymous substitutions between these genotypes. In contrast, no consistent deviation from neutrality was observed between intra-typic genotypes of EV-C96, CVA-21 and CVA-24 (Table 4).

**Table 4:** The summary of the results of MK-tests.

					Modified MK (Egea et al., 2008)		Modified MK (Bhatt et al., 2010)		
							Neutral site threshold frequency		
Clusters compared (N of strains in cluster)				MK (McDonald & Kreitman 1991)		low-frequency (<5%) variants excluded	0-<1	<0.50	0.05–0.75
EV-C96	(12)	CVA-21	(16)	***	***	***	***	***	***
EV-C96	(12)	CVA-24	(42)	***	***	***	***	***	***
EV-C96	(12)	EV-C99	(24)	***	***	***	***	***	***
EV-C96	(12)	EV-C95	(2)	***	***	***	*	*	*
CVA-21	(16)	CVA-24	(42)	***	***	***	***	***	***
CVA-21	(16)	EV-C99	(24)	***	***	***	***	***	***
EV-C95	(2)	CVA-21	(16)	***	***	***	***	***	***
EV-C95	(2)	EV-C99	(24)	***	***	***	***	***	***
EV-C95	(2)	CVA-24	(42)	***	***	***	***	***	***
EV-C99	(24)	CVA-24	(42)	***	***	***	***	***	***
EV-C96-A	(2)	EV-C96-B	(10)	-	-	-	-	-	-
EV-C96-A	(2)	EV-C96-B1	(3)	-	-	-	-	-	-
EV-C96-A	(2)	EV-C96-B2	(7)	-	-	-	-	-	-
EV-C96-B1	(3)	EV-C96-B2	(7)	-	-	-	-	-	-
CVA-21-A	(2)	CVA-21-B	(4)	-	-	-	-	-	-
CVA-21-A	(2)	CVA-21-C	(10)	-	-	-	-	-	-
CVA-21-B	(4)	CVA-21-C	(10)	*	-	-	-	-	-
EV-C99-A	(5)	EV-C99-B/C	(19)	***	***	***	***	**	***
EV-C99-A	(5)	EV-C99-B	(8)	***	***	***	***	***	***
EV-C99-A	(5)	EV-C99-C	(10)	***	**	*	-	-	-
EV-C99-B	(8)	EV-C99-C	(10)	-	-	-	-	-	-
CVA-24	(22)	CVA-24v	(20)	-	**	***	-	-	-
CVA-24-A	(2)	CVA-24-B	(4)	-	-	-	-	-	-
CVA-24-A	(2)	CVA-24-C	(2)	-	-	-	-	-	-
CVA-24-A	(2)	CVA-24-D	(9)	-	-	-	-	-	-
CVA-24-A	(2)	CVA-24-E	(2)	-	-	-	-	-	-
CVA-24-A	(2)	CVA-24-F	(2)	-	-	-	-	-	-
CVA-24-A	(2)	CVA-24v	(20)	-	-	-	-	-	*
CVA-24-B	(4)	CVA-24-C	(2)	-	-	-	-	-	-
CVA-24-B	(4)	CVA-24-D	(9)	-	-	-	*	-	*
CVA-24-B	(4)	CVA-24-E	(2)	-	-	-	-	-	-
CVA-24-B	(4)	CVA-24-F	(2)	-	-	-	-	-	-
CVA-24-B	(4)	CVA-24v	(20)	-	-	-	-	-	*
CVA-24-C	(2)	CVA-24-D	(9)	-	-	-	-	-	*
CVA-24-C	(2)	CVA-24-E	(2)	-	-	-	-	-	-
CVA-24-C	(2)	CVA-24-F	(2)	-	-	-	-	-	-
CVA-24-C	(2)	CVA-24v	(20)	-	-	-	-	-	-
CVA-24-D	(9)	CVA-24-E	(2)	-	*	*	-	-	-
CVA-24-D	(9)	CVA-24-F	(2)	-	-	-	-	-	-
CVA-24-D	(9)	CVA-24v	(20)	*	*	**	-	-	-
CVA-24-E	(2)	CVA-24-F	(2)	-	-	-	-	-	-
CVA-24-E	(2)	CVA-24v	(20)	-	-	-	-	-	*
CVA-24-F	(2)	CVA-24v	(20)	**	-	*	**	-	**
CVA-24-A	(2)	CVA-24-B-F/v	(40)	-	-	**	-	-	-
CVA-24-B/C	(6)	CVA-24-D-F/v	(35)	-	-	-	-	-	*
CVA-24-D-F	(15)	CVA-24v	(20)	-	-	*	-	-	-

Statistical significance for higher than expected proportion of fixed non-synonymous substitutions (i.e. nF/nP >> sF/sP) between different clusters is shown. P-values were calculated using chi-squared test. (* 0.05>P>0.01; ** 0.01>P>0.001; *** P<0.001; - not significant).

### Evolutionary rates

The rates of evolution and times of most recent common ancestors (tMRCA) were estimated for both the complete and the partial VP1 datasets using Bayesian MCMC method with relaxed molecular clock (Table 5). The estimated rates of substitution were 1.972×10^−3^ (high-probability distribution [95% HPD] range 1.097–2.903×10^−3^) and 2.839×10^−3^ (95% HPD range 2.177–3.509×10^−3^) substitutions/site/year for complete and partial VP1 datasets, respectively. The corresponding estimated rates of evolution and tMRCAs for each type in EV-C sub-cluster B are shown in the Table 5.

**Table 5:** The estimated mean rates of evolution (substitutions/site/year) and tMRCAs (year, BCE) for EV-C sub-cluster B partial VP1 sequence dataset.

Cluster	N (strains)	Mean rate (x 10^−3^)	1^st^ site	2^nd^ site	3^rd^ site	Coefficient of variation	Years isolated (range)	tMRCA (year)
**EV-C96**	36	**3.306** [0.472–5.949]	0.12 [0.16–0.23]	0.096 [0.075–0.12]	2.71 [2.67–2.74]	0.178 [4.642×10^−5^–0.31]	1998–2012	**1887** [1850–1920]
**CVA-21**	48	**3.139** [2.313–4.077]	0.16 [0.12–0.20]	0.056 [0.036–0.077]	2.78 [2.73–2.83]	0.304 [0.026–0.54]	1952–2010	**1843** [1784–1896]
**EV-C99**	60	**3.652** [0.930–6.469]	0.25 [0.22–0.28]	0.0975 [0.080–0.12]	2.66 [2.62–2.69]	0.23 [0.068–0.39]	1984–2011	**1814** [1753–1871]
**CVA-24**	118	**1.170** [0.993–2.424]	0.21 [0.19–0.24]	0.013 [0.12–0.15]	2.66 [2.63–2.68]	0.446 [0.307–0.589]	1952–2012	**1853** [1810–1890]
**EV-C sub-group B**	264	**2.839** [2.177–3.509]	**0.32** [0.28–0.35]	**0.23** [0.18–0.28]	**2.46** [2.40–2.52]	**0.404** [0.315–0.493]	1952–2012	**1548** [1385–1694]

The analysis was conducted using BEAST program. GTR model of substitution, lognormal relaxed clock and Bayesian Skyline demographic model were used in the analysis. The ranges of high-probability distribution [95% HPD] are given in brackets. EV-C95 was excluded from the table due to small number (2) of known isolates.

## Discussion

In order to elucidate the evolutionary processes that may lead to emergence of novel enterovirus types or genotypes, we determined evolutionary patterns of EV-C species sub-group B (i.e. strains of CVA-21, CVA-24, EV-C95, EV-C96 or EV-C99) VP1 gene using sequence data collected during entero/poliovirus surveillance together with sequence data retrieved from the GenBank.

### General aspects of molecular evolution and epidemiology of CVA-21, CVA-24, EV-C96 and EV-C99

Altogether 72 strains of EV-C sub-group B were sequenced in this study. Most of the strains were isolated in Finland during routine entero/poliovirus surveillance [23]. Previous reports have suggested low prevalence of EV-C viruses in temperate regions, whereas in tropical and sub-tropical regions EV-C viruses are highly prevalent [17], [18], [48]–[51]. In this study, EV-C strains were found regularly in Finland. However, in most cases the possibility of travel-related importation from tropical or sub-tropical regions cannot be excluded. Therefore, on the basis of this study it cannot be stated unambiguously whether the EV-C strains are capable of wide circulation in Finland. The majority of the strains showed no evidence of geographic or temporal clustering, suggesting no evidence for EV-C outbreaks in Finland. Although some lineages of EV-C99 and CVA-24 were detected during several years in Finland, repeated introduction from other countries cannot be excluded.

The mean nucleotide substitution rates of EV-C sub-group B (ranging from 1.170 to 3.625×10^−3^ with combined HPD interval of 1.170 to 3.625 for partial VP1) estimated using Bayesian MCMC method were slightly lower than the corresponding estimates for other enterovirus types (ranging from 4.2 to 8.6×10^−3^ for complete VP1 and 4.8 to 12×10^−3^ for partial VP1) [52]–[63]. Previously, similar low substitution rate has been estimated for partial VP1 coding region of CVA-24v strains using linear regression [64]. Correspondingly, the estimated tMRCAs of EV-C sub-group B dated earlier than those estimated for most other EV-types. Previously, correlation between the epidemiological fitness and higher mutation, genome replication and nucleotide substitution rates has been found with noroviruses [65]. Further studies should be conducted to assess if such correlation also applies for enteroviruses. However, it should be noted that the molecular clock analysis contains several limitations. For some of the types (e.g. EV-C96), only recently isolated strains are available. The estimated tMRCAs of the more distant ancestors of different EV-types (i.e. the deep nodes in the phylogenetic tree) are therefore highly unreliable since sequence data is available only from the most recent few decades. Likewise, the datasets analysed are likely to be biased due to sparse sampling and (both temporally and geographically) uneven sampling frequencies. Therefore, more sequence data, possibly from archived samples, would be needed to confirm the apparently lower rate of evolution in this EV-C sub-group.

### Evolutionary patterns within and between types

Each of the enterovirus types analysed in this study had relatively high intra-typic sequence diversities in VP1 (approaching the <25% nt divergence limit for grouping into the same type). The phylogenetic analysis of nucleotide sequences suggested robust grouping into intra-typic genotypes within each type. Within each type the vast majority of nucleotide substitutions were synonymous and the non-synonymous substitutions (i.e. amino acid substitutions) tended to cluster at distinct highly polymorphic sites. Signs of positive selection were detected in some of these highly polymorphic sites, while strong negative selection was indicated in most of the codons of VP1. Despite robust clustering to intra-typic genotypes, only few genotype-specific ‘signature’ amino acids were detected between these intra-typic clusters, whereas several amino acid sites showed evidence of parallel evolution (i.e. similar amino acid substitutions had apparently occurred independently in different genetic lineages).

In contrast, when *different* types were compared, the McDonald-Kreitman tests suggested a clear tendency towards fixation of type-specific ‘signature’ amino acids. Furthermore, several type-specific insertions/deletions were detected and the locations of highly polymorphic or positively selected amino acid sites overlapped only partially between different types. These results suggest that permanent fixation of type-specific amino acids seems to be a hallmark associated with evolution of *different* enterovirus types, whereas neutral evolution and/or (most likely frequency-dependent, see below) positive selection in few highly polymorphic amino acid sites were the dominant forms of evolution when strains *within* a type were compared. An exception to the rarity of permanently fixed signature amino acids in most intra-typic lineages was EV-C99 genotype A that showed similar differences (i.e. fixation of ‘signature’ amino acids and insertion/deletion sites) to those detected in inter-typic comparisons. The strains of EV-C99 genotype A also had relatively low pairwise similarities (a minimum of 73.1% nucleotide and 86.1% amino acid similarity) with some of the strains of EV-C99 genotypes B and C. Curiously, EV-C99 groups A and B/C may also have antigenic differences since the strains of genotype A show cross-neutralization with the antibodies raised against CVA-24-Joseph whereas the strains of EV-C99 group B do not show such cross-neutralization [5]. Therefore, the EV-C99 genotypes A and B/C may be under a process of divergent evolution that might ultimately lead to two distinct virus types. Further complete genome sequencing is needed to evaluate whether the strains of EV-C99 genotype A are divergent enough to merit classification to a separate type.

The different evolutionary patterns within and between EV types may also have implication on the genetic classification of enteroviruses. In the current classification scheme, EV-strains are classified into the same type, if they have more than 75% nucleotide and more than 85% (or 88%) amino acid similarities in the VP1 region and into different types, if the strains that have less than 70% nucleotide and 85% amino acid similarities [3], [5]. However, divergent strains that have pairwise nt/aa similarities in the ‘grey-zone’ of current typing (i.e. 70–75% nt and/or 85–88% aa similarity) are detected regularly. The hallmarks of inter-typic comparisons (fixation of type-specific amino acids and insertion/deletion sites) could be applied as an additional classification criterion in such cases. In this study, EV-C99 and CVA-24 were clearly separated on the basis of MK-tests despite the ‘grey-zone’ nucleotide/amino acid similarities between these types. However, further studies on other enterovirus species should be conducted to study the universality of these potential novel classification criteria first.

### Possible structural constraints in intra-typic evolution

Within each EV-type analysed, most of the codons in VP1 were detected to be under negative selection. This suggests a strong evolutionary pressure to retain the amino acid sequence and, thus, the structure of VP1. On the basis of the structure of CVA-21 [42], the highly polymorphic amino acid sites are most likely located in the loops between beta-sheets and the structurally disordered amino- and carboxyl-terminal segments of VP1. Such pattern may be explained by frequency-dependent selection (a rare variant has greater fitness than a common variant) posed by the host immune system to amino acids at the virus surface (e.g., antigenic sites). While a mutation at antigenic site could allow the virus to escape from the host immune response, due to the adaptability of the host immune system, the advantage could be short term. At the virus type-level, this changing selective pressure could result in a highly polymorphic site. Alternatively, neutral amino acid substitutions located in the surface loops or amino- and carboxy-terminal segments may have remained in the virus population due to genetic drift.

Similar results (i.e. high proportion of negatively selected codons combined with neutral evolution and/or evidence of positive selection in few codons) have been detected also for other EV-types including PV [66]–[68], E-30 [69], EV-71 [56], CVA-4 [58], E-6 [57] and CVB-5 [61]. These results suggest that the intra-typic evolution of the VP1 protein is most likely dominated by (mostly neutral) synonymous mutations combined with frequency-dependent selection and/or neutral evolution at antigenic sites. Intra-typic lineages would therefore have similar structural constraints with variation occurring predominantly at specific sites.

Interestingly, the AHC-causing CVA-24v strains showed less amino acid diversity in VP1 than the other CVA-24 strains. Furthermore, the amino acid polymorphisms were located in different sites in CVA-24v compared to the non-AHC-causing CVA-24 strains. The AHC-causing variants of CVA-24 emerged in Singapore in the year 1970 [21]. Both epidemiological and phylogenetic studies suggest a single origin for CVA-24v strains [5], [21]. Therefore, it is likely that CVA-24v strains have undergone a strict population bottle-neck during the colonisation of a new target tissue (i.e. conjunctiva). The high degree of amino acid conservation between CVA-24v strains may be due to this population bottle-neck. Alternatively, CVA-24v strains might be under more strict negative selection than the other CVA-24 lineages. However, the overall dN/dS values were similar for both CVA-24v and the non-AHC-causing CVA-24 genotypes suggesting similar strength of negative selection for both groups.In contrast to the intra-typic evolution, the inter-typic comparisons between EV-C96, CVA-21, EV-99 and CVA-24 suggest that mere frequency-dependent selection in antigenic sites is not sufficient to explain the differences between the EV types. Apparently, the evolution leading to a formation of a new enterovirus type has included permanent fixation and insertion/deletion of distinct amino acids. Furthermore, the polymorphic sites overlap only partially between different EV types. These observations suggest that, in addition to amino acid changes at antigenic sites, also structural changes may have occurred during divergent evolution of EV-types. Hypothetically, a structural change could expose new amino acid sites to selective pressure imposed by the host immune system and, on the other hand, require fixation at sites where polymorphism was previously allowed. Furthermore, an (fitness decreasing) amino acid mutation may require compensatory amino acid fixation(s) elsewhere in the capsid protein.

Alternatively, fixation of neutral amino acid substitutions may have occurred in the ancestral populations of EV-types due to genetic drift during population bottle-necks. However, although the estimated tMRCAs of the more distant ancestors of different EV-types (i.e. the deep nodes in the phylogenetic tree) are highly unreliable, it is likely that the type-specific amino acids have remained conserved for hundreds of years. This suggests strong selection pressures favouring these type-specific amino acids in the given genetic context. Therefore, it is more likely that the permanent fixation of type-specific amino acids is due to dissimilar selection pressures subjected to the ancestor lineages of different enterovirus types. Further studies are needed to elucidate the processes leading to fixation of type-specific amino acid substitutions.

### Concluding remarks

The sequence analysis presented in this article suggests different modes of evolution within enterovirus types (resulting in intra-typic lineages) and during evolution, which leads to larger scale (type-specific) differences. In this respect, intra-typic genetic change would be dominated by silent mutations accompanied by amino acid polymorphism occurring dominantly at immunogenic sites. This genetic change can be observed as a high rate of synonymous mutations, strong negative selection and amino acid polymorphism and/or positive selection at distinct sites in structurally disordered regions. Inter-typic differences, on the other hand, included permanent fixation and insertion/deletion of distinct ‘signature’-amino acids that could be a result of larger scale changes in the capsid structure.

## Supporting Information

Figure S1 The capsid pentamer of CVA-21; top view (a, d and g) and side view (b, c, e, f, h and i). VP1 is shown in blue, VP2 in green, VP3 in red and VP4 in yellow. The VP1 amino acids that showed type-specific fixation between CVA-21 and EV-C96 (a-c), CVA-21 and EV-C99 (d–f) or CVA-21 and CVA-24 (g-i) are shown in white. The amino acids that showed evidence of positive selection within EV-C96 (a–c) or EV-C99 (d–f) are shown in cyan. The amino acids that showed evidence of positive selection within CVA-24v cluster or in non-AHC-causing strains are shown in purple and orange, respectively (g–i).
(PDF)

Table S1 The numbers of sites in the MK-test classes (s = synonymous; n = non-synonymous; F = fixed; P = polymorphic). The numbers were calculated using standard MacDonald-Kreitman test [36]. P-values were calculated with chi-squared test (* 0.05>P>0.01; ** 0.01>P>0.001; *** P<0.001; NS =  not significant).
(DOCX)

Table S2 The numbers of sites in the McDonald-Kreitman test classes (s = synonymous; n = non-synonymous; F = fixed; P = polymorphic). The numbers were calculated using modified MacDonald-Kreitman test [38] with Jukes-Cantor substitution model. P-values were calculated with chi-squared test (* 0.05>P>0.01; ** 0.01>P>0.001; *** P<0.001; NS  =  not significant).
(DOCX)

Table S3 The numbers of sites in the McDonald-Kreitman test classes (s = synonymous; n = non-synonymous; F = fixed; P = polymorphic). The numbers were calculated using modified MacDonald-Kreitman test [38] with Jukes-Cantor substitution model. Low-frequency variants (<5%) were excluded from the analysis. P-values were calculated with chi-squared test (* 0.05>P>0.01; ** 0.01>P>0.001; *** P<0.001; NS =  not significant).
(DOCX)

Table S4 The numbers of sites in the McDonald-Kreitman test classes (s = synonymous; n = non-synonymous; F = fixed; P = polymorphic). The numbers were calculated using modified MacDonald-Kreitman test [39]. Neutral class site frequency thresholds of 0.0–1.0 were used in the analysis. P-values were calculated with chi-squared test (* 0.05>P>0.01; ** 0.01>P>0.001; *** P<0.001; NS  =  not significant).
(DOCX)

Table S5 The numbers of sites in the MK-test classes (s = synonymous; n = non-synonymous; F = fixed; P = polymorphic). The numbers were calculated using modified MacDonald-Kreitman test [39]. Neutral class site frequency thresholds of 0.0–0.5 [40] were used in the analysis. P-values were calculated with chi-squared test (* 0.05>P>0.01; ** 0.01>P>0.001; *** P<0.001; NS =  not significant).
(DOCX)

Table S6 The numbers of sites in the MK-test classes (s = synonymous; n = non-synonymous; F = fixed; P = polymorphic). The numbers were calculated using modified MacDonald-Kreitman test [39]. Neutral class site frequency thresholds of 0.05–0.75 were used in the analysis. P-values were calculated with chi-squared test (* 0.05>P>0.01; ** 0.01>P>0.001; *** P<0.001; NS  =  not significant).
(DOCX)
