# Supplementary figures and images for: The Evolution of Vp1 Gene in Enterovirus C Species Sub-Group That Contains Types CVA-21, CVA-24, EV-C95, EV-C96 and EV-C99
Source: PLoS One. 2014 Apr 2;9(4):e93737. doi: 10.1371/journal.pone.0093737 (PMC3973639; doi:10.1371/journal.pone.0093737)

## CVA-21 vs.EV-C96

**A**

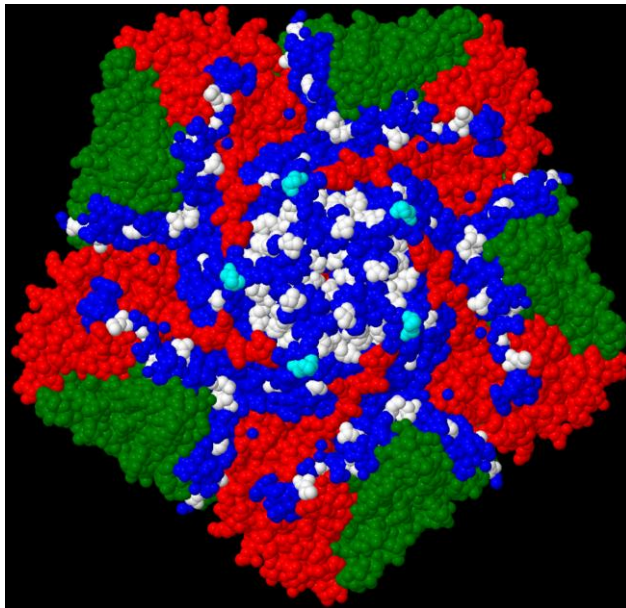

**B**

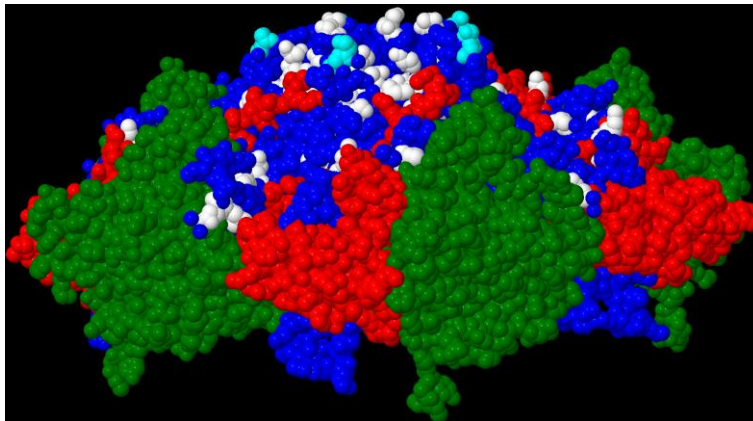

**C**

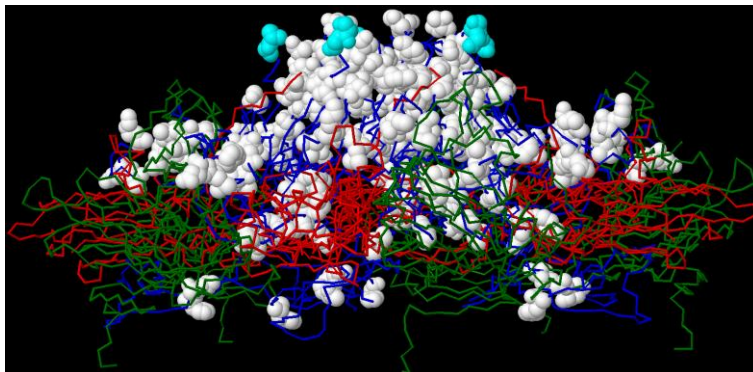

## CVA-21 vs.EV-C99

D

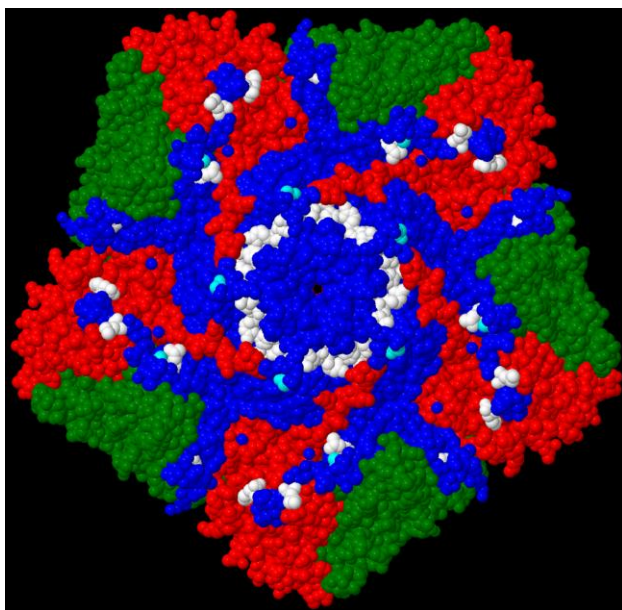

E

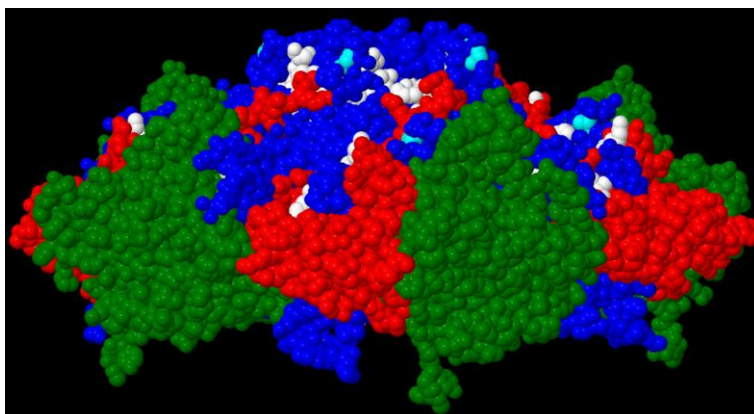

F

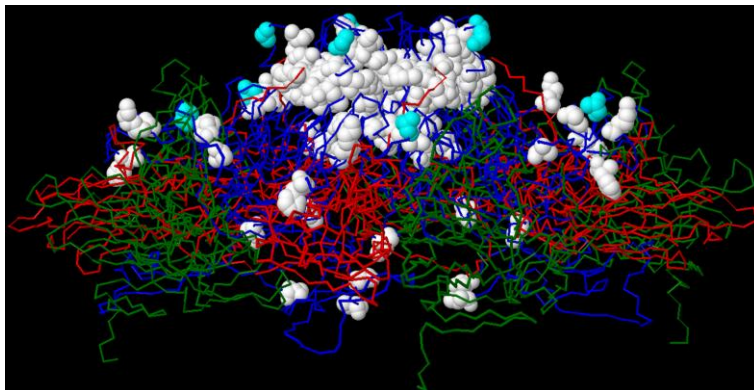

## CVA-21 vs.CVA-24

G

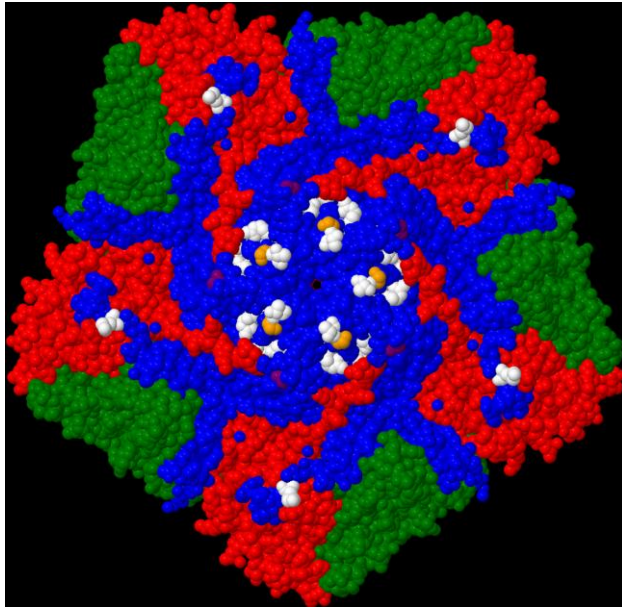

H

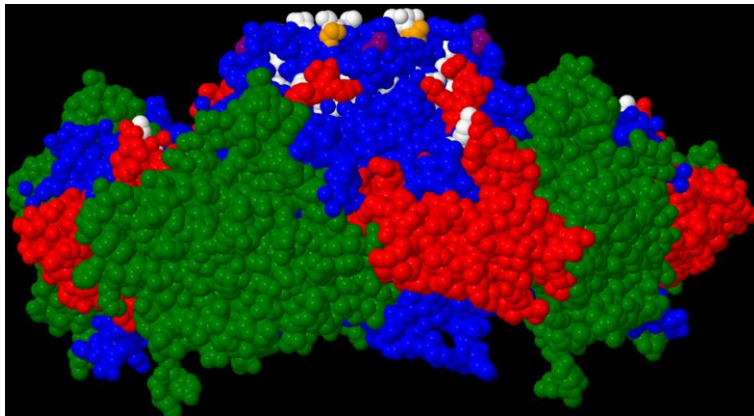

I

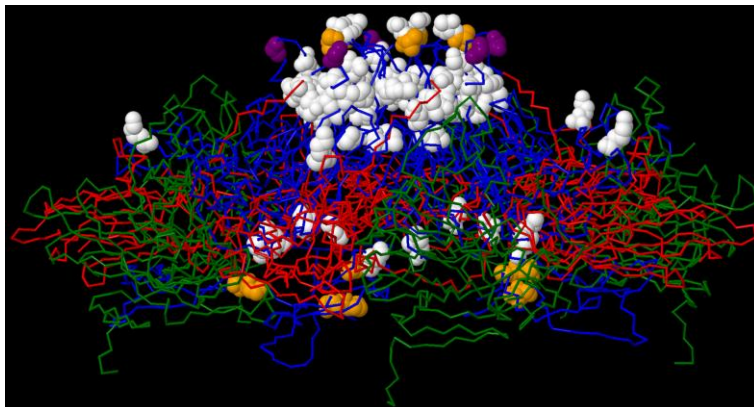

Supplement: Figure S1 — The capsid pentamer of CVA-21; top view (a, d and g) and side view (b, c, e, f, h and i). VP1 is shown in blue, VP2 in green, VP3 in red and VP4 in yellow. The VP1 amino acids that showed type-specific fixation between CVA-21 and EV-C96 (a-c), CVA-21 and EV-C99 (d–f) or CVA-21 and CVA-24 (g-i) are shown in white. The amino acids that showed evidence of positive selection within EV-C96 (a–c) or EV-C99 (d–f) are shown in cyan. The amino acids that showed evidence of positive selection within CVA-24v cluster or in non-AHC-causing strains are shown in purple and orange, respectively (g–i). (PDF) [file pone.0093737.s001.pdf]
