# Supplementary material for: The Evolution of Vp1 Gene in Enterovirus C Species Sub-Group That Contains Types CVA-21, CVA-24, EV-C95, EV-C96 and EV-C99
Source: PLoS One. 2014 Apr 2;9(4):e93737. doi: 10.1371/journal.pone.0093737 (PMC3973639; doi:10.1371/journal.pone.0093737)
Supplement: Table S1 — The numbers of sites in the MK-test classes (s = synonymous; n = non-synonymous; F = fixed; P = polymorphic). The numbers were calculated using standard MacDonald-Kreitman test [36]. P-values were calculated with chi-squared test (* 0.05>P>0.01; ** 0.01>P>0.001; *** P<0.001; NS = not significant). (DOCX) [file pone.0093737.s002.docx]

**Table S1.** The numbers of sites in the MK-test classes (s=synonymous; n=non-synonymous; F=fixed; P=polymorphic). The numbers were calculated using standard MacDonald-Kreitman test [36]. P-values were calculated with chi-squared test (* 0.05 > P > 0.01; ** 0.01 > P > 0.001; *** P < 0.001; NS = not significant).

| **Clusters compared** | | **Fixed differences (between groups)** | |  | **Polymorphic** | | **p-value** |
| --- | --- | --- | --- | --- | --- | --- | --- |
|  |  | **sF** | **nF** |  | **sP** | **nP** |  |
| **EV-C96** | **CVA-21** | 10 | 87 |  | 469 | 37 | *** |
| **EV-C96** | **CVA-24** | 8 | 42 |  | 433 | 41 | *** |
| **EV-C96** | **EV-C99** | 7 | 36 |  | 395 | 27 | *** |
| **EV-C96** | **EV-C95** | 62 | 103 |  | 357 | 31 | *** |
| **CVA-21** | **CVA-24** | 4 | 39 |  | 438 | 35 | *** |
| **CVA-21** | **EV-C99** | 4 | 38 |  | 401 | 23 | *** |
| **CVA-21** | **EV-C95** | 45 | 47 |  | 409 | 20 | *** |
| **EV-C99** | **CVA-24** | 1 | 9 |  | 365 | 25 | *** |
| **EV-C99** | **EV-C95** | 24 | 50 |  | 370 | 15 | *** |
| **CVA-24** | **EV-C95** | 22 | 50 |  | 407 | 30 | *** |
| **EV-C96-A** | **EV-C96-B** | 18 | 1 |  | 379 | 49 | NS |
| **EV-C96-A** | **EV-C96-B1** | 63 | 9 |  | 279 | 46 | NS |
| **EV-C96-A** | **EV-C96-B2** | 39 | 7 |  | 325 | 47 | NS |
| **EV-C96-B1** | **EV-C96-B2** | 20 | 3 |  | 341 | 39 | NS |
| **CVA-21-A** | **CVA-21-B** | 98 | 6 |  | 269 | 15 | NS |
| **CVA-21-A** | **CVA-21-C** | 91 | 13 |  | 270 | 18 | NS |
| **CVA-21-B** | **CVA-21-C** | 92 | 14 |  | 230 | 15 | * |
| **EV-C99-A** | **EV-C99-B/C** | 10 | 14 |  | 431 | 35 | *** |
| **EV-C99-A** | **EV-C99-B** | 19 | 22 |  | 460 | 43 | *** |
| **EV-C99-A** | **EV-C99-C** | 105 | 76 |  | 267 | 59 | *** |
| **EV-C99-B** | **EV-C99-C** | 25 | 3 |  | 384 | 51 | NS |
| **CVA-24** | **CVA-24v** | 1 | 1 |  | 427 | 36 | NS |
| **CVA-24-A** | **CVA-24-B** | 45 | 5 |  | 322 | 35 | NS |
| **CVA-24-A** | **CVA-24-C** | 76 | 10 |  | 217 | 34 | NS |
| **CVA-24-A** | **CVA-24-D** | 36 | 9 |  | 345 | 34 | * |
| **CVA-24-A** | **CVA-24-E** | 76 | 17 |  | 228 | 35 | NS |
| **CVA-24-A** | **CVA-24-F** | 116 | 26 |  | 110 | 17 | NS |
| **CVA-24-A** | **CVA-24v** | 63 | 16 |  | 292 | 42 | NS |
| **CVA-24-B** | **CVA-24-C** | 37 | 7 |  | 319 | 35 | NS |
| **CVA-24-B** | **CVA-24-D** | 26 | 6 |  | 400 | 44 | NS |
| **CVA-24-B** | **CVA-24-E** | 40 | 10 |  | 333 | 38 | NS |
| **CVA-24-B** | **CVA-24-F** | 74 | 11 |  | 259 | 29 | NS |
| **CVA-24-B** | **CVA-24v** | 24 | 3 |  | 382 | 77 | NS |
| **CVA-24-C** | **CVA-24-D** | 36 | 2 |  | 346 | 38 | NS |
| **CVA-24-C** | **CVA-24-E** | 72 | 9 |  | 245 | 38 | NS |
| **CVA-24-C** | **CVA-24-F** | 121 | 11 |  | 136 | 22 | NS |
| **CVA-24-C** | **CVA-24v** | 60 | 9 |  | 298 | 48 | NS |
| **CVA-24-D** | **CVA-24-E** | 21 | 5 |  | 357 | 36 | NS |
| **CVA-24-D** | **CVA-24-F** | 53 | 6 |  | 298 | 28 | NS |
| **CVA-24-D** | **CVA-24v** | 28 | 9 |  | 363 | 45 | * |
| **CVA-24-E** | **CVA-24-F** | 107 | 13 |  | 152 | 23 | NS |
| **CVA-24-E** | **CVA-24v** | 50 | 11 |  | 312 | 49 | NS |
| **CVA-24-F** | **CVA-24v** | 62 | 21 |  | 237 | 32 | ** |
| **CVA-24-A** | **CVA-24-B-F/v** | 7 | 0 |  | 403 | 30 | NS |
| **CVA-24-B/C** | **CVA-24-D-F/v** | 4 | 0 |  | 421 | 37 | NS |
| **CVA-24-D-F** | **CVA-24v** | 8 | 2 |  | 400 | 43 | NS |
