# Supplementary material for: The Evolution of Vp1 Gene in Enterovirus C Species Sub-Group That Contains Types CVA-21, CVA-24, EV-C95, EV-C96 and EV-C99
Source: PLoS One. 2014 Apr 2;9(4):e93737. doi: 10.1371/journal.pone.0093737 (PMC3973639; doi:10.1371/journal.pone.0093737)
Supplement: Table S2 — The numbers of sites in the McDonald-Kreitman test classes (s = synonymous; n = non-synonymous; F = fixed; P = polymorphic). The numbers were calculated using modified MacDonald-Kreitman test [38] with Jukes-Cantor substitution model. P-values were calculated with chi-squared test (* 0.05>P>0.01; ** 0.01>P>0.001; *** P<0.001; NS = not significant). (DOCX) [file pone.0093737.s003.docx]

**Table S2.** The numbers of sites in the McDonald-Kreitman test classes (s=synonymous; n=non-synonymous; F=fixed; P=polymorphic). The numbers were calculated using modified MacDonald-Kreitman test [38] with Jukes-Cantor substitution model. P-values were calculated with chi-squared test (* 0.05 > P > 0.01; ** 0.01 > P > 0.001; *** P < 0.001; NS = not significant).

| **Clusters compared** | | **Fixed changes (between groups)** | |  | **Polymorphic** | | **p-value (chi-square)** |
| --- | --- | --- | --- | --- | --- | --- | --- |
|  |  | **sF** | **nF** |  | **sP** | **nP** |  |
| **EV-C96** | **CVA-21** | 25.95 | 102.74 |  | 938 | 122 | *** |
| **EV-C96** | **CVA-24** | 24.73 | 47.07 |  | 1113 | 251 | *** |
| **EV-C96** | **EV-C99** | 16.81 | 42.74 |  | 1103 | 237 | *** |
| **EV-C96** | **EV-C95** | 117.5 | 105.22 |  | 474 | 86 | *** |
| **CVA-21** | **CVA-24** | 16.84 | 73.16 |  | 952 | 93 | *** |
| **CVA-21** | **EV-C99** | 4.04 | 65.04 |  | 1105 | 163 | *** |
| **CVA-21** | **EV-C95** | 63.88 | 52.65 |  | 496 | 40 | *** |
| **EV-C99** | **CVA-24** | 3.02 | 14.19 |  | 1274 | 292 | *** |
| **EV-C99** | **EV-C95** | 36.79 | 69.63 |  | 643 | 128 | *** |
| **CVA-24** | **EV-C95** | 41.84 | 67.29 |  | 647 | 144 | *** |
| **EV-C96-A** | **EV-C96-B** | 27.05 | 2.00 |  | 550 | 101 | NS |
| **EV-C96-A** | **EV-C96-B1** | 85.33 | 6.03 |  | 329 | 56 | NS |
| **EV-C96-A** | **EV-C96-B2** | 46.94 | 7.04 |  | 447 | 79 | NS |
| **EV-C96-B1** | **EV-C96-B2** | 21.28 | 3.00 |  | 468 | 79 | NS |
| **CVA-21-A** | **CVA-21-B** | 153.5 | 4.01 |  | 300 | 15 | NS |
| **CVA-21-A** | **CVA-21-C** | 132.37 | 13.17 |  | 309 | 21 | NS |
| **CVA-21-B** | **CVA-21-C** | 146.47 | 10.10 |  | 267 | 18 | NS |
| **EV-C99-A** | **EV-C99-B/C** | 15.70 | 17.29 |  | 836 | 143 | *** |
| **EV-C99-A** | **EV-C99-C** | 59.22 | 23.53 |  | 510 | 100 | ** |
| **EV-C99-A** | **EV-C99-B** | 27.02 | 19.36 |  | 731 | 105 | *** |
| **EV-C99-C** | **EV-C99-B** | 30.59 | 1.00 |  | 573 | 119 | NS |
| **CVA-24** | **CVA-24v** | 2.01 | 3.00 |  | 859 | 170 | ** |
| **CVA-24-A** | **CVA-24-B** | 57.88 | 3.00 |  | 386 | 70 | NS |
| **CVA-24-A** | **CVA-24-C** | 100.82 | 10.09 |  | 234 | 35 | NS |
| **CVA-24-A** | **CVA-24-D** | 41.75 | 11.11 |  | 423 | 62 | NS |
| **CVA-24-A** | **CVA-24-E** | 100.73 | 17.28 |  | 251 | 36 | NS |
| **CVA-24-A** | **CVA-24-F** | 195.43 | 26.67 |  | 112 | 17 | NS |
| **CVA-24-A** | **CVA-24v** | 78.69 | 17.28 |  | 343 | 56 | NS |
| **CVA-24-B** | **CVA-24-C** | 46.97 | 4.01 |  | 414 | 75 | NS |
| **CVA-24-B** | **CVA-24-D** | 33.04 | 2.00 |  | 603 | 102 | NS |
| **CVA-24-B** | **CVA-24-E** | 50.96 | 8.06 |  | 431 | 76 | NS |
| **CVA-24-B** | **CVA-24-F** | 108.45 | 9.07 |  | 292 | 57 | NS |
| **CVA-24-B** | **CVA-24v** | 56.44 | 7.04 |  | 523 | 96 | NS |
| **CVA-24-C** | **CVA-24-D** | 40.47 | 3.00 |  | 451 | 67 | NS |
| **CVA-24-C** | **CVA-24-E** | 93.65 | 9.07 |  | 279 | 41 | NS |
| **CVA-24-C** | **CVA-24-F** | 212.09 | 11.11 |  | 140 | 22 | NS |
| **CVA-24-C** | **CVA-24v** | 73.99 | 9.07 |  | 371 | 61 | NS |
| **CVA-24-D** | **CVA-24-E** | 22.41 | 8.06 |  | 468 | 68 | * |
| **CVA-24-D** | **CVA-24-F** | 63.58 | 8.06 |  | 329 | 49 | NS |
| **CVA-24-D** | **CVA-24v** | 30.60 | 10.09 |  | 560 | 88 | * |
| **CVA-24-E** | **CVA-24-F** | 168.81 | 13.16 |  | 157 | 23 | NS |
| **CVA-24-E** | **CVA-24v** | 59.18 | 12.14 |  | 388 | 62 | NS |
| **CVA-24-F** | **CVA-24v** | 78.69 | 21.43 |  | 249 | 43 | NS |
| **CVA-24-A** | **CVA-24-B-F/v** | 8.19 | 0 |  | 731 | 169 | NS |
| **CVA-24-B/C** | **CVA-24-D-F/v** | 4.04 | 0 |  | 922 | 192 | NS |
| **CVA-24-D-F** | **CVA-24v** | 9.24 | 3.00 |  | 706 | 128 | NS |
