# Supplementary material for: The Evolution of Vp1 Gene in Enterovirus C Species Sub-Group That Contains Types CVA-21, CVA-24, EV-C95, EV-C96 and EV-C99
Source: PLoS One. 2014 Apr 2;9(4):e93737. doi: 10.1371/journal.pone.0093737 (PMC3973639; doi:10.1371/journal.pone.0093737)
Supplement: Table S3 — The numbers of sites in the McDonald-Kreitman test classes (s = synonymous; n = non-synonymous; F = fixed; P = polymorphic). The numbers were calculated using modified MacDonald-Kreitman test [38] with Jukes-Cantor substitution model. Low-frequency variants (<5%) were excluded from the analysis. P-values were calculated with chi-squared test (* 0.05>P>0.01; ** 0.01>P>0.001; *** P<0.001; NS = not significant). (DOCX) [file pone.0093737.s004.docx]

**Table S3.** The numbers of sites in the McDonald-Kreitman test classes (s=synonymous; n=non-synonymous; F=fixed; P=polymorphic). The numbers were calculated using modified MacDonald-Kreitman test [38] with Jukes-Cantor substitution model. Low-frequency variants (< 5%) were excluded from the analysis. P-values were calculated with chi-squared test (* 0.05 > P > 0.01; ** 0.01 > P > 0.001; *** P < 0.001; NS = not significant).

| **Clusters compared** | | **Fixed changes (between groups)** | |  | **Polymorphic** | | **p-value**  **(chi-square)** |
| --- | --- | --- | --- | --- | --- | --- | --- |
|  |  | **sF** | **nF** |  | **sP** | **nP** |  |
| **EV-C96** | **CVA-21** | 100.3 | 30.7 |  | 939 | 122 | *** |
| **EV-C96** | **CVA-24** | 38.07 | 60.44 |  | 917 | 141 | *** |
| **EV-C96** | **EV-C99** | 27.12 | 28.24 |  | 945 | 156 | *** |
| **EV-C96** | **EV-C95** | 125.88 | 102.76 |  | 474 | 86 | *** |
| **CVA-21** | **CVA-24** | 11.38 | 60.48 |  | 1111 | 180 | *** |
| **CVA-21** | **EV-C99** | 15.73 | 73.12 |  | 921 | 77 | *** |
| **CVA-21** | **EV-C95** | 63.88 | 52.65 |  | 497 | 40 | *** |
| **EV-C99** | **CVA-24** | 13.55 | 25.65 |  | 919 | 106 | *** |
| **EV-C99** | **EV-C95** | 81.30 | 75.50 |  | 489 | 58 | *** |
| **CVA-24** | **EV-C95** | 87.94 | 83.67 |  | 456 | 41 | *** |
| **EV-C96-A** | **EV-C96-B** | 27.05 | 2.00 |  | 550 | 101 | NS |
| **EV-C96-A** | **EV-C96-B1** | 85.33 | 6.03 |  | 329 | 56 | NS |
| **EV-C96-A** | **EV-C96-B2** | 46.94 | 7.04 |  | 447 | 79 | NS |
| **EV-C96-B1** | **EV-C96-B2** | 21.28 | 3.00 |  | 468 | 79 | NS |
| **CVA-21-A** | **CVA-21-B** | 158.64 | 3.00 |  | 300 | 15 | NS |
| **CVA-21-A** | **CVA-21-C** | 132.37 | 13.17 |  | 309 | 21 | NS |
| **CVA-21-B** | **CVA-21-C** | 146.47 | 10.10 |  | 267 | 18 | NS |
| **EV-C99-A** | **EV-C99-B/C** | 15.70 | 19.29 |  | 842 | 140 | *** |
| **EV-C99-A** | **EV-C99-B** | 27.02 | 19.36 |  | 738 | 102 | *** |
| **EV-C99-A** | **EV-C99-C** | 62.07 | 22.49 |  | 514 | 98 | * |
| **EV-C99-B** | **EV-C99-C** | 30.59 | 1.00 |  | 580 | 116 | NS |
| **CVA-24** | **CVA-24v** | 9.25 | 6.03 |  | 631 | 69 | *** |
| **CVA-24-A** | **CVA-24-B** | 65.08 | 1.00 |  | 386 | 70 | NS |
| **CVA-24-A** | **CVA-24-C** | 100.82 | 10.09 |  | 234 | 35 | NS |
| **CVA-24-A** | **CVA-24-D** | 41.75 | 11.11 |  | 423 | 62 | NS |
| **CVA-24-A** | **CVA-24-E** | 100.73 | 17.28 |  | 251 | 36 | NS |
| **CVA-24-A** | **CVA-24-F** | 195.43 | 26.67 |  | 112 | 17 | NS |
| **CVA-24-A** | **CVA-24v** | 122.49 | 21.44 |  | 240 | 25 | NS |
| **CVA-24-B** | **CVA-24-C** | 50.98 | 3.00 |  | 414 | 75 | NS |
| **CVA-24-B** | **CVA-24-D** | 33.04 | 2.00 |  | 603 | 102 | NS |
| **CVA-24-B** | **CVA-24-E** | 53.68 | 7.04 |  | 431 | 76 | NS |
| **CVA-24-B** | **CVA-24-F** | 114.25 | 8.06 |  | 292 | 57 | NS |
| **CVA-24-B** | **CVA-24v** | 80.65 | 8.06 |  | 420 | 65 | NS |
| **CVA-24-C** | **CVA-24-D** | 44.33 | 2.00 |  | 451 | 67 | NS |
| **CVA-24-C** | **CVA-24-E** | 93.65 | 9.07 |  | 279 | 41 | NS |
| **CVA-24-C** | **CVA-24-F** | 212.09 | 11.11 |  | 140 | 22 | NS |
| **CVA-24-C** | **CVA-24v** | 114.41 | 12.14 |  | 268 | 30 | NS |
| **CVA-24-D** | **CVA-24-E** | 22.41 | 8.06 |  | 468 | 68 | * |
| **CVA-24-D** | **CVA-24-F** | 63.58 | 8.06 |  | 329 | 49 | NS |
| **CVA-24-D** | **CVA-24v** | 47.01 | 14.19 |  | 457 | 57 | ** |
| **CVA-24-E** | **CVA-24-F** | 168.81 | 13.16 |  | 157 | 23 | NS |
| **CVA-24-E** | **CVA-24v** | 97.43 | 17.28 |  | 285 | 31 | NS |
| **CVA-24-F** | **CVA-24v** | 128.84 | 25.48 |  | 146 | 43 | NS |
| **CVA-24-A** | **CVA-24-B-F/v** | 20.17 | 8.06 |  | 530 | 55 | ** |
| **CVA-24-B/C** | **CVA-24v** | 7.15 | 0 |  | 830 | 124 | NS |
| **CVA-24-D-F** | **CVA-24v** | 14.61 | 6.03 |  | 603 | 97 | * |
