# Supplementary material for: The Evolution of Vp1 Gene in Enterovirus C Species Sub-Group That Contains Types CVA-21, CVA-24, EV-C95, EV-C96 and EV-C99
Source: PLoS One. 2014 Apr 2;9(4):e93737. doi: 10.1371/journal.pone.0093737 (PMC3973639; doi:10.1371/journal.pone.0093737)
Supplement: Table S4 — The numbers of sites in the McDonald-Kreitman test classes (s = synonymous; n = non-synonymous; F = fixed; P = polymorphic). The numbers were calculated using modified MacDonald-Kreitman test [39]. Neutral class site frequency thresholds of 0.0–1.0 were used in the analysis. P-values were calculated with chi-squared test (* 0.05>P>0.01; ** 0.01>P>0.001; *** P<0.001; NS = not significant). (DOCX) [file pone.0093737.s005.docx]

**Table S4.** The numbers of sites in the McDonald-Kreitman test classes (s=synonymous; n=non-synonymous; F=fixed; P=polymorphic). The numbers were calculated using modified MacDonald-Kreitman test [39]. Neutral class site frequency thresholds of 0.0-1.0 were used in the analysis. P-values were calculated with chi-squared test (* 0.05 > P > 0.01; ** 0.01 > P > 0.001; *** P < 0.001; NS = not significant).

| **Clusters compared** | |  | **Fixed differences (between groups)** | |  | **Polymorphic** | | **p-value** |
| --- | --- | --- | --- | --- | --- | --- | --- | --- |
| **Ancestral** | **Main** |  | **sF** | **nF** |  | **sP** | **nP** |  |
| **EV-C96** | **CVA-21** |  | 25.17 | 119.50 |  | 231.83 | 68.5 | *** |
| **EV-C96** | **CVA-24** |  | 7.67 | 68.0 |  | 266.33 | 140.0 | *** |
| **EV-C96** | **EV-C99** |  | 8.67 | 59.33 |  | 258.33 | 138.67 | *** |
| **EV-C96** | **EV-C95** |  | 162.5 | 151.5 |  | 13.5 | 2.5 | * |
| **CVA-21** | **CVA-24** |  | 11.33 | 62.5 |  | 259.67 | 127.5 | *** |
| **CVA-21** | **EV-C99** |  | 9.50 | 64.83 |  | 255.50 | 127.17 | *** |
| **EV-C95** | **CVA-21** |  | 22.67 | 56.16 |  | 258.33 | 51.83 | *** |
| **EV-C95** | **CVA-24** |  | 257.17 | 128.17 |  | 14.83 | 59.83 | *** |
| **EV-C95** | **EV-C99** |  | 14.17 | 62.67 |  | 252.83 | 128.33 | *** |
| **EV-C99** | **CVA-24** |  | 6.5 | 29.0 |  | 279.5 | 129.0 | *** |
| **EV-C96-A** | **EV-C96-B** |  | 20.33 | 9.83 |  | 254.67 | 67.17 | NS |
| **EV-C96-A** | **EV-C96-B1** |  | 76.83 | 17.0 |  | 151.17 | 27.0 | NS |
| **EV-C96-A** | **EV-C96-B2** |  | 40.33 | 15.5 |  | 216.67 | 48.5 | NS |
| **EV-C96-B1** | **EV-C96-B2** |  | 43.0 | 10.0 |  | 215.0 | 48.0 | NS |
| **CVA-21-A** | **CVA-21-B** |  | 123.0 | 9.5 |  | 105.0 | 5.5 | NS |
| **CVA-21-A** | **CVA-21-C** |  | 114.5 | 17.5 |  | 110.5 | 13.5 | NS |
| **CVA-21-B** | **CVA-21-C** |  | 102.5 | 17.83 |  | 111.5 | 13.17 | NS |
| **EV-C99-A** | **EV-C99-B/C** |  | 19.83 | 22.17 |  | 262.16 | 90.83 | *** |
| **EV-C99-A** | **EV-C99-C** |  | 94.33 | 31.0 |  | 136.67 | 51.0 | NS |
| **EV-C99-A** | **EV-C99-B** |  | 29.33 | 27.0 |  | 244.67 | 62.0 | *** |
| **EV-C99-B** | **EV-C99-C** |  | 66.5 | 8.5 |  | 153.5 | 50.5 | NS |
| **CVA-24** | **CVA-24v** |  | 43.67 | 14.0 |  | 207.33 | 41.0 | NS |
| **CVA-24-A** | **CVA-24-B** |  | 56.17 | 9.0 |  | 201.83 | 58.0 | NS |
| **CVA-24-A** | **CVA-24-C** |  | 101.5 | 15.5 |  | 117.5 | 21.5 | NS |
| **CVA-24-A** | **CVA-24-D** |  | 35.5 | 11.5 |  | 241.5 | 47.5 | NS |
| **CVA-24-A** | **CVA-24-E** |  | 92.5 | 22.5 |  | 126.5 | 23.5 | NS |
| **CVA-24-A** | **CVA-24-F** |  | 145.5 | 36.0 |  | 8.5 | 2.0 | NS |
| **CVA-24-A** | **CVA-24v** |  | 66.0 | 22.5 |  | 197.0 | 40.5 | NS |
| **CVA-24-B** | **CVA-24-C** |  | 91.5 | 17.0 |  | 116.5 | 23.0 | NS |
| **CVA-24-B** | **CVA-24-D** |  | 36.5 | 17.0 |  | 235.5 | 49.0 | * |
| **CVA-24-B** | **CVA-24-E** |  | 88.5 | 23.5 |  | 122.5 | 23.5 | NS |
| **CVA-24-B** | **CVA-24-F** |  | 146.0 | 26.0 |  | 9.0 | 2.0 | NS |
| **CVA-24-B** | **CVA-24v** |  | 64.67 | 24.0 |  | 194.33 | 42.0 | NS |
| **CVA-24-C** | **CVA-24-D** |  | 37.16 | 12.5 |  | 241.83 | 45.5 | NS |
| **CVA-24-C** | **CVA-24-E** |  | 91.5 | 18.5 |  | 130.5 | 22.5 | NS |
| **CVA-24-C** | **CVA-24-F** |  | 155.0 | 27.0 |  | 9.0 | 2.0 | NS |
| **CVA-24-C** | **CVA-24v** |  | 57.83 | 17.5 |  | 199.16 | 40.5 | NS |
| **CVA-24-D** | **CVA-24-E** |  | 79.5 | 16.0 |  | 125.5 | 24.0 | NS |
| **CVA-24-D** | **CVA-24-F** |  | 146.0 | 18.0 |  | 9.0 | 2.0 | NS |
| **CVA-24-D** | **CVA-24v** |  | 49.83 | 16.0 |  | 205.16 | 41.0 | NS |
| **CVA-24-E** | **CVA-24-F** |  | 144.5 | 25.0 |  | 8.5 | 2.0 | NS |
| **CVA-24-E** | **CVA-24v** |  | 58.5 | 19.5 |  | 205.5 | 38.5 | NS |
| **CVA-24-F** | **CVA-24v** |  | 48.33 | 21.5 |  | 208.67 | 39.5 | ** |
| **CVA-24-A** | **CVA-24-B-F/v** |  | 4.33 | 0.0 |  | 287.67 | 129.0 | NS |
| **CVA-24-B/C** | **CVA-24-D-F/v** |  | 8.83 | 7.0 |  | 284.17 | 101.0 | NS |
| **CVA-24-D-F** | **CVA-24v** |  | 47.5 | 15.5 |  | 205.5 | 41.5 | NS |
